# The Nature of Diamino Linker and Halogen Bonding Define Selectivity of Pyrrolopyrimidine-Based LIMK1 Inhibitors

**DOI:** 10.3389/fchem.2021.781213

**Published:** 2021-12-13

**Authors:** Daryl Ariawan, Carol Au, Esmeralda Paric, Thomas Fath, Yazi D. Ke, Michael Kassiou, Janet van Eersel, Lars M. Ittner

**Affiliations:** ^1^ Dementia Research Centre, Department of Biomedical Science, Faculty of Medicine and Health Sciences, Macquarie University, Sydney, NSW, Australia; ^2^ School of Chemistry, The University of Sydney, Darlington, NSW, Australia

**Keywords:** LIMK, kinase inhibitor, SAR, cofilin phosphorylation, actin cytoskeleton

## Abstract

The LIM-domain kinase (LIMK) family consists of two isoforms, LIMK1 and LIMK2, which are highly homologous, making selective inhibitor development challenging. LIMK regulates dynamics of the actin cytoskeleton, thereby impacting many cellular functions including cell morphology and motility. Here, we designed and synthesised analogues of a known pyrrolopyrimidine LIMK inhibitor with moderate selectivity for LIMK1 over LIMK2 to gain insights into which features contribute to both activity and selectivity. We incorporated a different stereochemistry around a cyclohexyl central moiety to achieve better selectivity for different LIMK isoforms. Inhibitory activity was assessed by kinase assays, and biological effects in cells were determined using an *in vitro* wound closure assay. Interestingly, a slight change in stereochemistry alters LIMK isoform selectivity. Finally, a docking study was performed to predict how the new compounds interact with the target.

## Introduction

The LIM-domain kinase (LIMK) are a family of serine/threonine protein kinases that act downstream of Rho GTPases. Cofilin, the known LIMK substrate, is a key regulator on actin skeleton dynamics (Bernard 2007; Scott and Olson 2007). LIMKs phosphorylate cofilin at the Ser3 position. Once phosphorylated, cofilin can no longer bind to actin, leading to the accumulation of actin polymers (Tanaka et al., 2018; Ben Zablah et al., 2020). Aside from actin cytoskeletal regulation, LIMKs also play an important role in microtubule organisation (Bernard 2007; Scott and Olson 2007; Prunier et al., 2017).

The increased activity of the LIMK1 isoform has been associated with several diseases including Alzheimer’s diseases (AD) (Heredia et al., 2006; Piccioli and Littleton 2014), cancer (Davila et al., 2007; Kang et al., 2021; Shi et al., 2021), and HIV (Vorster et al., 2011; Wen et al., 2014). One of the hallmarks of AD is characterised by deposition of the β-amyloid peptide (Aβ) (Murphy and Levine 2010). Aβ deposition has a detrimental effect on actin cytoskeleton, and recent studies indicated involvement of the Rho-GTPase pathway (Rush et al., 2018). LIMK1 inhibition has shown protection against Aβ toxicity in primary neurons and mice (Heredia et al., 2006; Henderson et al., 2019).

Inhibition of LIMK1 also shows beneficial effects in cancer. Accordingly, overexpression of LIMK1 in breast cancer cell lines MCF-7 and MDA-MB-231 increased their motility, while inhibition of LIMK1 attenuated this effect (Yoshioka et al., 2003). Targeting LIMK1 in lung cancer cells inhibits cell proliferation and induces apoptosis (Zhang et al., 2021). Thus, LIMK1 is a promising drug target, potentially for a range of diseases.

**FIGURE 1:**
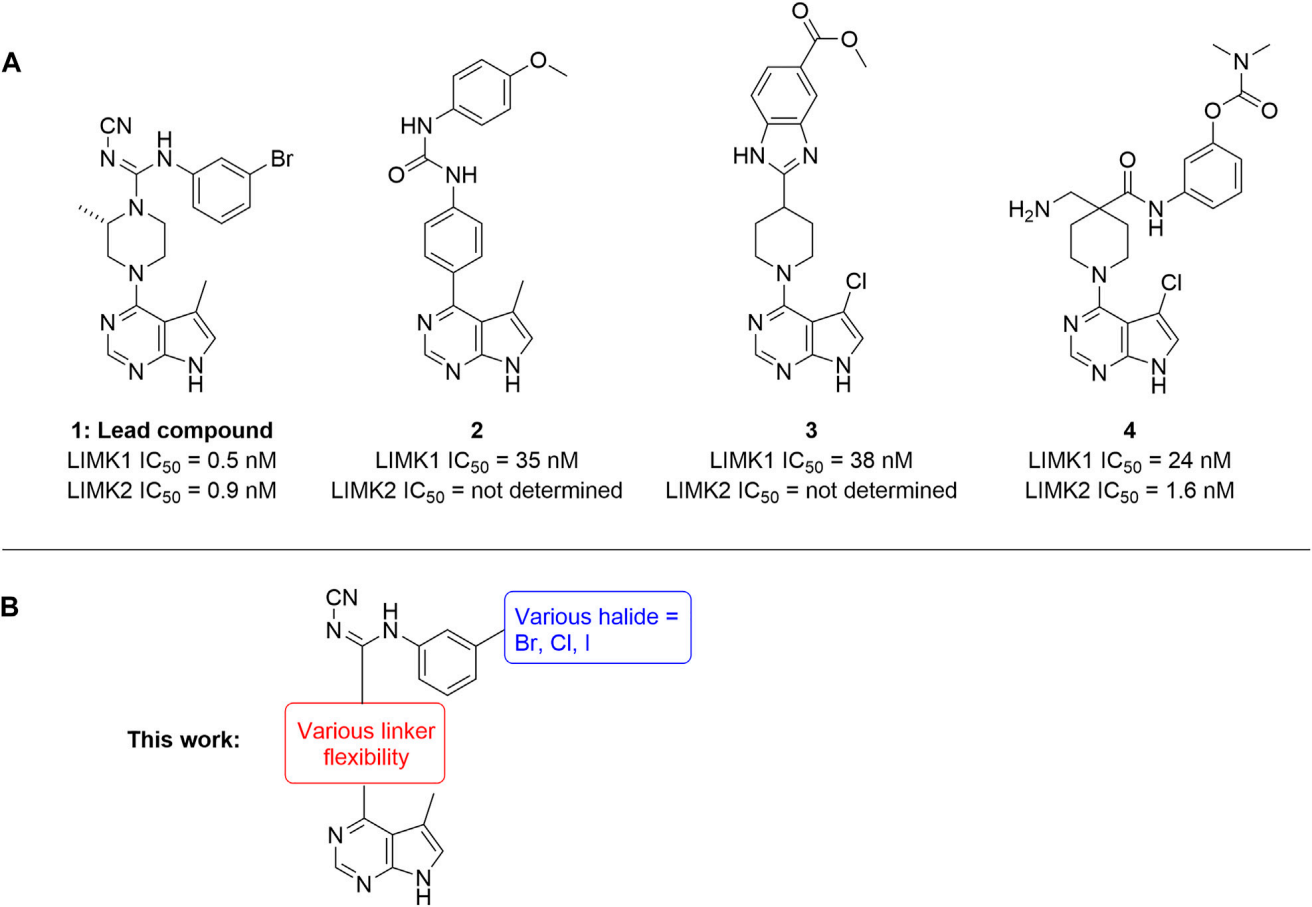
**(A)** Structure of known pyrrolopyrimidine LIMK inhibitor and their inhibitor profile. **(B)** Scaffold modification in this work.

**FIGURE 2:**
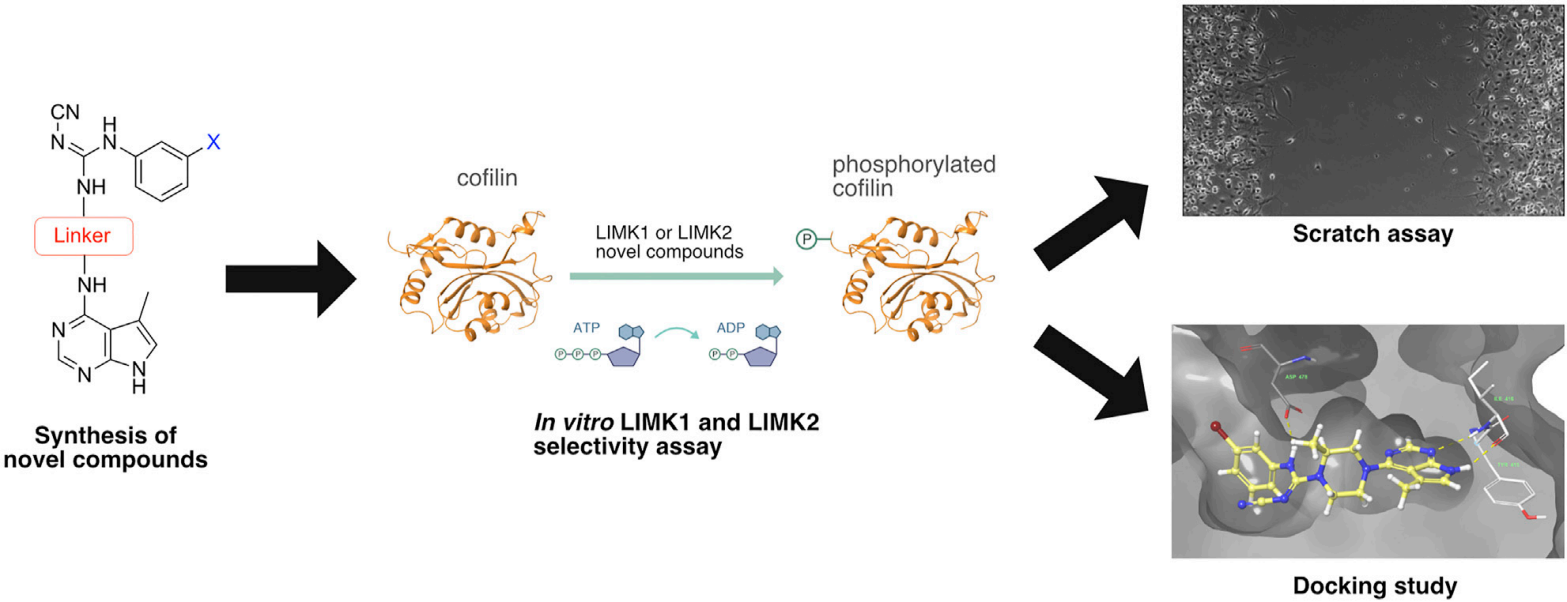
Workflow of this study.

Several LIMK1 inhibitors based on the pyrrolopyrimidine scaffold have been reported (Figure 1) (Harrison et al., 2009; Boland et al., 2015; Yin et al., 2015; Yi et al., 2017). Compound **1** was reported as part of the development of potent LIMK2 inhibitors (Harrison et al., 2009) but has a slightly higher activity against LIMK1. Accordingly, compound **1** was reported to have an IC_50_ of 0.5 and 0.9 nM for inhibiting LIMK1 and LIMK2, respectively (Harrison et al., 2009). In this work, we report modifications of the central piperidine linker motif to explore the effects of conformational flexibility and halogen exchange of the phenyl cyanoguanidine motif to probe LIMK activity and selectivity (Figure 1B). We tested the compounds against LIMK1 and LIMK2 to explore the selectivity and their effect on cellular based assay (Figure 2).

## Results and Discussion

**SCHEME 1:**
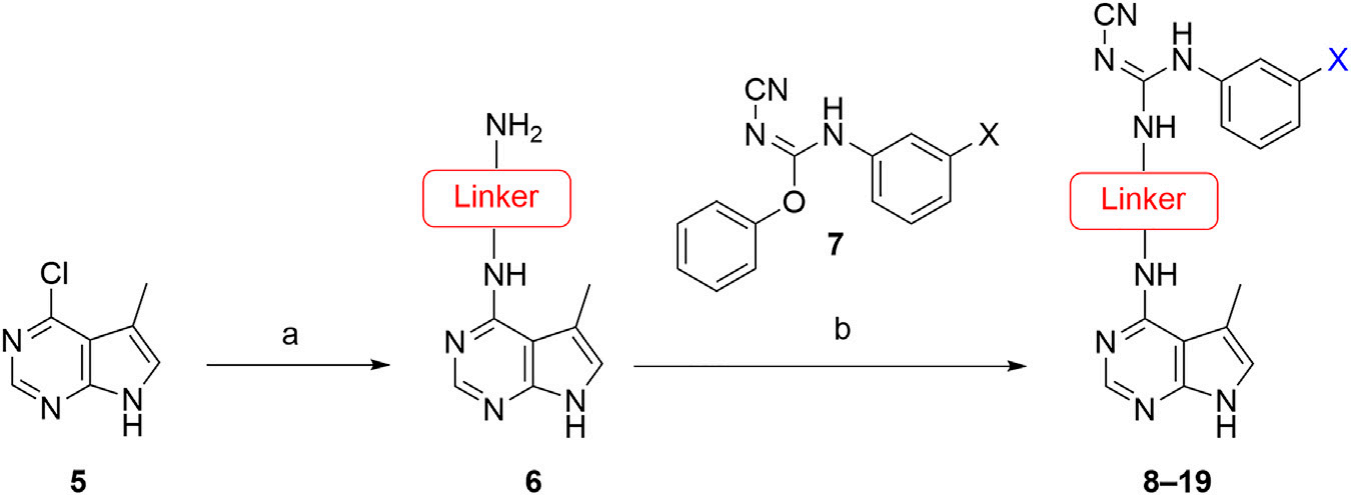
General synthetic procedure. Reagents and condition: **(A)** diaminoalkane linker, diisopropylethylamine, isopropanol, reflux, 16 h, 50–97%; **(B)** cyanoguanidine **7**, triethylamine, MeCN, reflux, 16 h, 16%–39%.

The approach for investigating different amine linkers is described in Scheme 1. Briefly, the nucleophilic substitution of chloropyrrolopyrimidine (**5**) with various diamino alkane linkers afforded the desired amine derivatives (**6**) (Scheme 1). These amines were then converted to target compounds **8–19** by reaction with a range of halogen-substituted phenyl cyanocarbamamimidates (**7**), (Scheme 1) (Harrison et al., 2009). A structure–activity relationship for compounds **8–19** was initially established by measuring inhibition against LIMK1 at a concentration of 0.1 μM for each compound (Table 1).

**TABLE 1:** Synthesis of initial investigation of linker chain and halide substitutions.

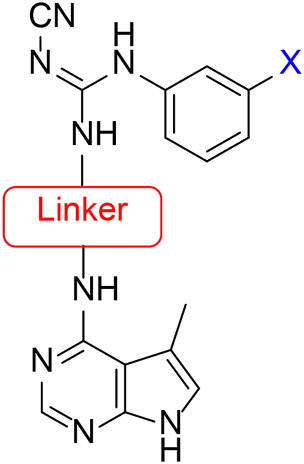			
**Compound**	**Amine linker**	**X =**	**% LIMK1 inhibition at 0.1 μM^a^**
1	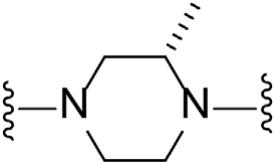	Br	94.3 ± 0.2
8	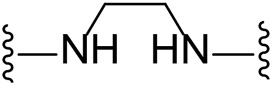	Cl	-^b^
9	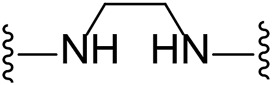	Br	29.4 ± 10.3
10	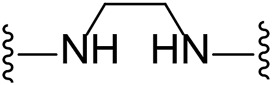	I	17.8 ± 7.1
11	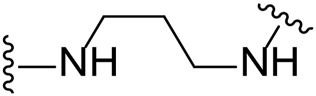	Cl	15.0 ± 10.7
12	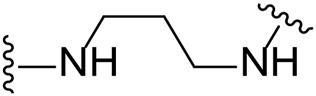	Br	-^b^
13	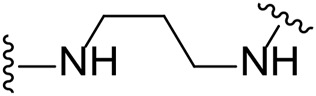	I	17.8 ± 7.1
14	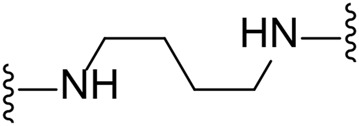	Cl	-^b^
15	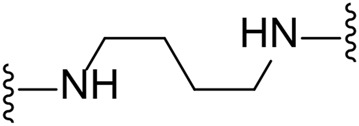	Br	47.1 ± 1.6
16	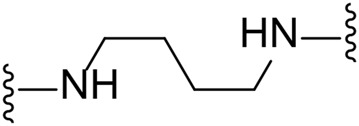	I	41.0 ± 2.5
17	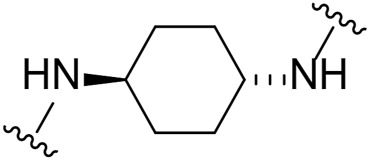	Cl	41.7 ± 0.1
18	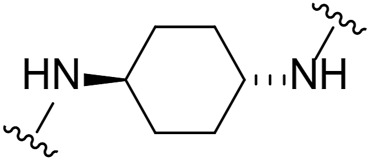	Br	69.2 ± 1.7
**Compound**	**Amine linker**	**X =**	**% LIMK1 inhibition at 0.1 μM^a^**
19	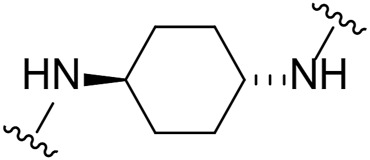	I	66.6 ± 8.8

^a^Measured in Promega ADP-Glo™ kinase Assay system.

b No inhibition at 0.1 uM.

Substituting the piperidine linker motif in **1** with an ethyl linker (**9**) decreased inhibitory activity at LIMK1 by threefold when assayed at 0.1 μM despite the similar distance between the two nitrogens in the linker (Table 1). Increasing the linker chain to a propyl motif (**12**) abolished the inhibitory activity while the longer butyl linker (**15**) restored the inhibitory activity to 47%. Incorporating the conformationally restricted 1,4-diaminocyclohexane linker (**18**) increased the inhibitory activity to 69%. Thus, it can be implied that the ring linker is needed for hydrophobic interaction within the binding pocket.

We next turned our attention to the halogen at the phenyl cyanoguanidine motif. Chloride analogues with ethyl and butyl linkers (**8** and **14**, respectively) completely abolished inhibitory activity. The propyl linker, chloride analogue **11**, displayed a minimal inhibitory effect of 15%, while with the 1,4-cyclohexyl linker, the chloride analogue **17** decreased the inhibitory activity compared to the bromide analogue **18** from 69% to 42%, respectively. Iodide substitution with the ethyl linker (**7**) showed a twofold decrease in inhibitory activity compared to **9**. Using the propyl linker motif, iodide analogue **13** gave a similar inhibitory profile to chloride analogue **11**. For the butyl and cyclohexyl linkers, iodide substitution (**16** and **19,** respectively) gave similar potencies to bromide analogues (**15** and **18**).

**SCHEME 2:**
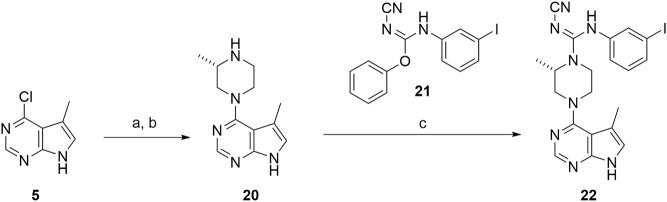
Synthesis of iodide analogues of compound **1.** Reagents and condition: **(A)** *(S)*-tert-butyl 2-methylpiperazine-1-carboxylate, diisopropylethylamine, isopropanol, reflux, 16 h; **(B)** 50% TFA in DCM, rt, 3 h, 90% over two steps; **(C)** cyanoguanidine **21**, triethylamine, MeCN, reflux, 16 h, 8%.

Given the LIMK1 inhibition of the cyclohexyl analogues **17–19**, we next investigated the amino 1,2-diaminocyclohexyl analogues **23–26** (Table 2). We were furthermore interested to see the effects of changing bromide to iodide in the lead compound **1**. Compound **22**, the iodide analogue of **1**, was synthesised in a similar fashion (Scheme 2).

**TABLE 2:** Inhibition profile of diaminocyclohexyl linker analogues.
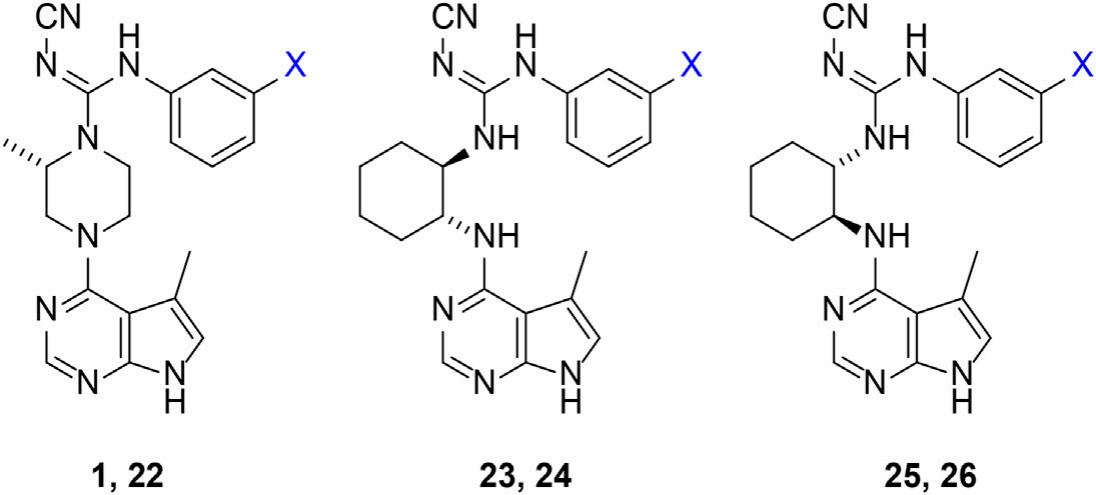

Compound	X =	% LIMK1 inhibition at 0.1 μM^a^	IC_50_ (nM)^b^	
			LIMK1	LIMK2

1	Br	94.3 ± 0.2	0.28 ± 0.01	0.86 ± 0.05
22	I	95.5 ± 0.4	0.13 ± 0.01	0.47 ± 0.01
23	Br	96.7 ± 0.1	2,830 ± 37	2,651 ± 15
24	I	93.2 ± 1.2	5,083 ± 14	3,380 ± 19
25	Br	96.0 ± 0.5	47%^c^	>10 μM
26	I	94.9 ± 0.8	8,360 ± 21	>10 μM

a Measured in Promega ADP-Glo™ kinase Assay system.

b Measured on the transfer of^33^P-labelled phosphate from ATP, to the kinase substrate (cofilin) by Reaction Biology Corporation.

c Inhibition at 10 μM.

Our *in-house* measurement of LIMK1 inhibition resulted in >90% inhibition at 0.1 μM for all the 1,2-diaminocyclohexyl analogues (**23–26**) (Table 2). Similar results were also achieved with the lead compound (**1**) and its iodide analogue (**22**). Given this high potency toward LIMK1, we next investigated the selectivity profile of these compounds. LIMK1 and LIMK2 exhibit the same domain architecture and have overlapping substrate specificities. While targeting LIMK1 has been used *in vivo* in cancer (Zhang et al., 2021) and AD mouse models (Henderson et al., 2019), LIMK2 functionality is essential for proper functionality of the eye (Rice et al., 2012) and in spermatogenesis (Takahashi et al., 2002). Thus, a specific LIMK1 isoform inhibition is desirable.

To measure the selectivity against LIMK isoforms, the radiotracer assay measuring the transfer of ^33^P from ATP to the substrate was used since there was no ADP-Glo™ kinase assay system available for LIMK2 (Table 2). Substitution of bromide in compound **1** to iodide in compound **22** resulted in a twofold potency increase for both LIMK1 and LIMK2. Compounds **23–26** were significantly less potent than compounds **1** and **22**. Interestingly, however, the stereochemistry of the cyclohexyl linker changed the inhibitory bias toward LIMK1 over LIMK2. Compounds **25** and **26** bearing the (*R, R*)-1,2-cyclohexyl linker were selective toward LIMK1, with no significant inhibition of LIMK2. For comparison, compounds **23** and **24** bearing the (*S, S*)-1,2-cyclohexyl linker had a slightly higher potency toward LIMK2 than LIMK1.

### Scratch Assay

**FIGURE 3:**
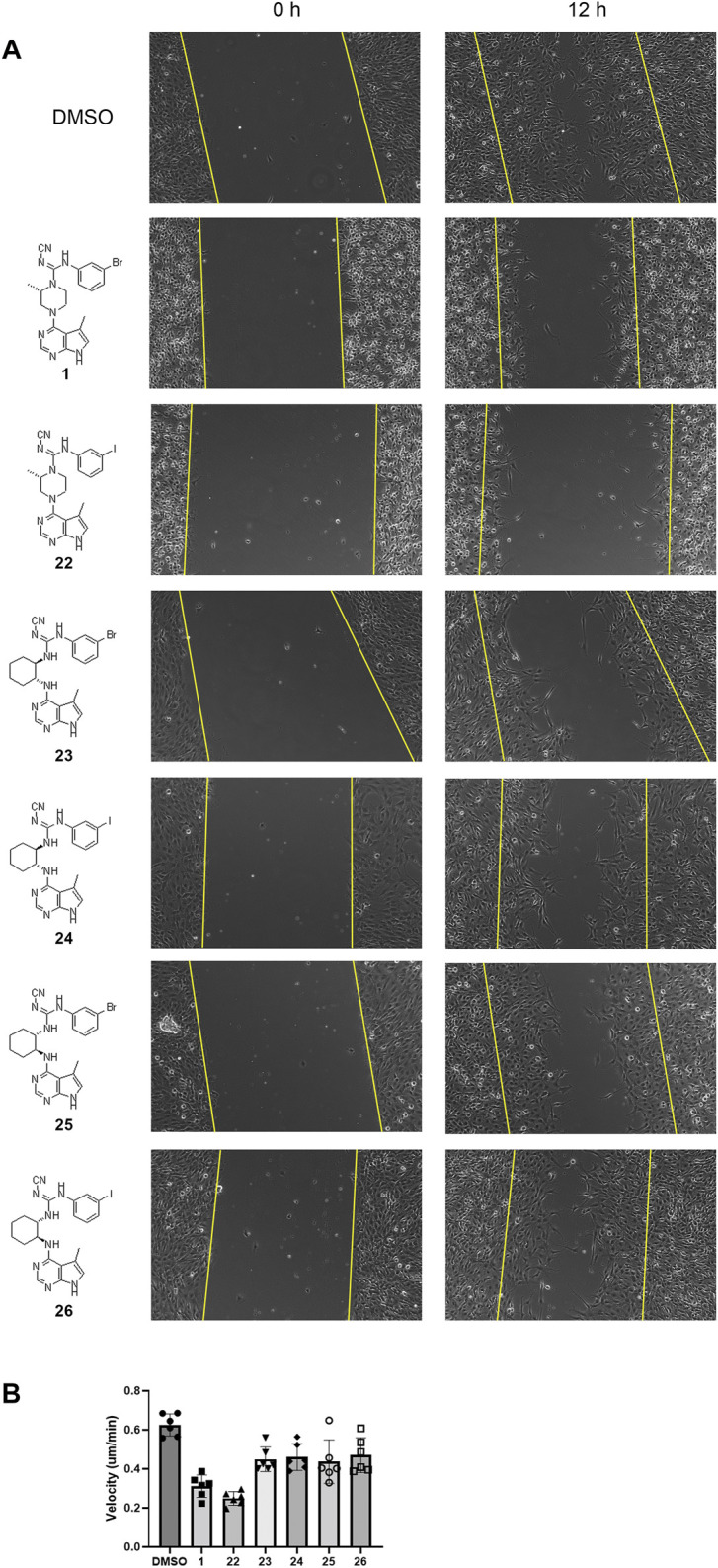
Delayed wound closure in scratch assays upon LIMK inhibition. **(A)** Representative scratch assay images at 0 and 12 h for compounds **1** and **22–26**. Yellow lines indicate the edges of the scratch wound and therefore starting point of cell migration. **(B)** Quantification of cell velocity from six independent experiments using single-cell tracing.

Cofilin, the substrate for LIMK1, regulates the actin skeleton and thus regulates cell migration (Bravo-Cordero et al., 2013). To understand the inhibitory effect of compounds **1** and **22–26** *in vitro*, scratch assays were performed. The artificial wound was created by “scratching” in the middle of the well with confluent C6 cells in culture. Live imaging was captured for 24 h to measure the distance of cell migration (Figure 3; Supplementary video).

All compounds tested in the scratch assay showed significant reduction in cell migration velocity. Among the cyclohexyl analogues **23–26**, there is no significant difference in the cell velocity. The lead compound **1** reduced the cell velocity by twofold compared to the untreated cell. Substitution from bromide to iodide in compound **22** lowered the migration even further. Overall, the cell migration velocity reflects the IC_50_ value measured by the radiotracer assay (Reaction Biology Corporation). Thus, it can be implied that these inhibitors work by hindering the binding of ATP into LIMK.

### Docking Study

Docking studies were performed for piperazine and cyclohexyl analogues to rationalise the selectivity trends against LIMK1 and LIMK2. A published crystal structure of LIMK1 (PDB: 5NXC) (Mathea et al., 2017) and LIMK2 (PDB: 4TPT) (Goodwin et al., 2015) bound with inhibitor formed the basis for our docking study, which we performed using Schrodinger GLIDE. Re-docking the ligand from the crystal structure generated a binding pose with RMSD <2.0 Å, providing confidence that the docking protocol is reliable (Supplementary Figures S1, S2).

**FIGURE 4:**
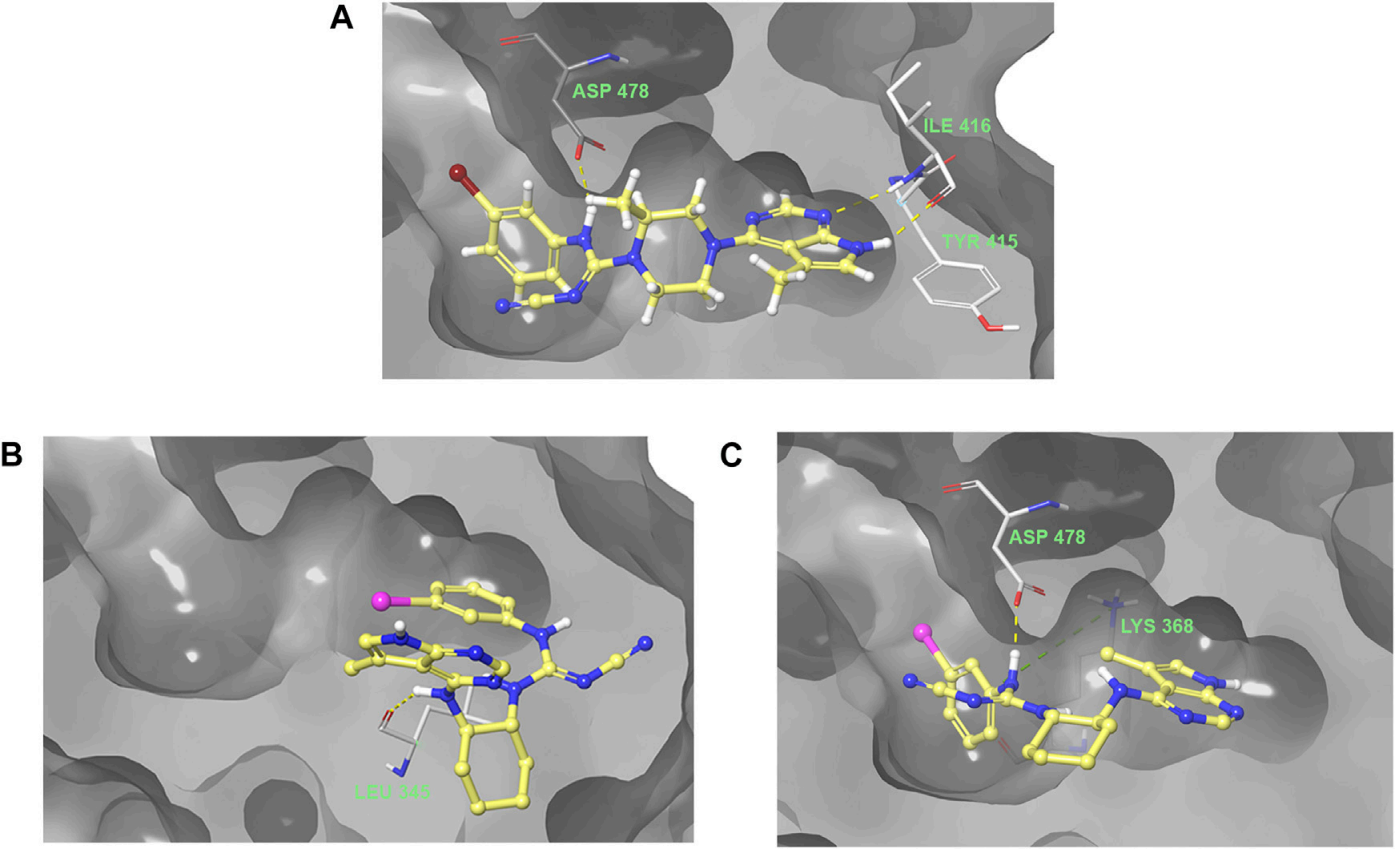
Predicted binding poses of ligand **1 (A)**, **24 (B)**, and **26 (C)** into the ATP-binding pocket of LIMK1.

The predicted binding pose of **1** to LIMK1 is shown in Figure 4. The key interaction of compound **1** includes hydrogen bonding of pyrrolopyrimidine moiety to Ile 416 and hydrogen bonding of cyanoguanidine moiety to Asp 478 of LIMK1. Hydrogen bonding with Ile 416 is detrimental for this potency as shown by previous structure–activity studies (Cui et al., 2015; Yin et al., 2015). It is important to note that the hydrophobic interaction around piperazine and aryl moieties also contributes to high potency of compound **1**.

Incorporation of the 1,2-diamino cyclohexyl motif changed the 3D structure of the ligand and thus changed the binding pose into the ATP-binding pocket of LIMK1. Docking of ligand **24** resulted in a binding pose with the iodophenyl moiety facing toward the inside of the binding pocket. Compound **26** maintained the hydrogen bonding of the cyanoguanidine moiety to Asp 478 while losing the hydrogen bonding to Ile 416 due to the rigidity of the cyclohexyl group. The absence of hydrogen bonding into Ile 416 could be the reason for the reduction in potency.

**FIGURE 5:**
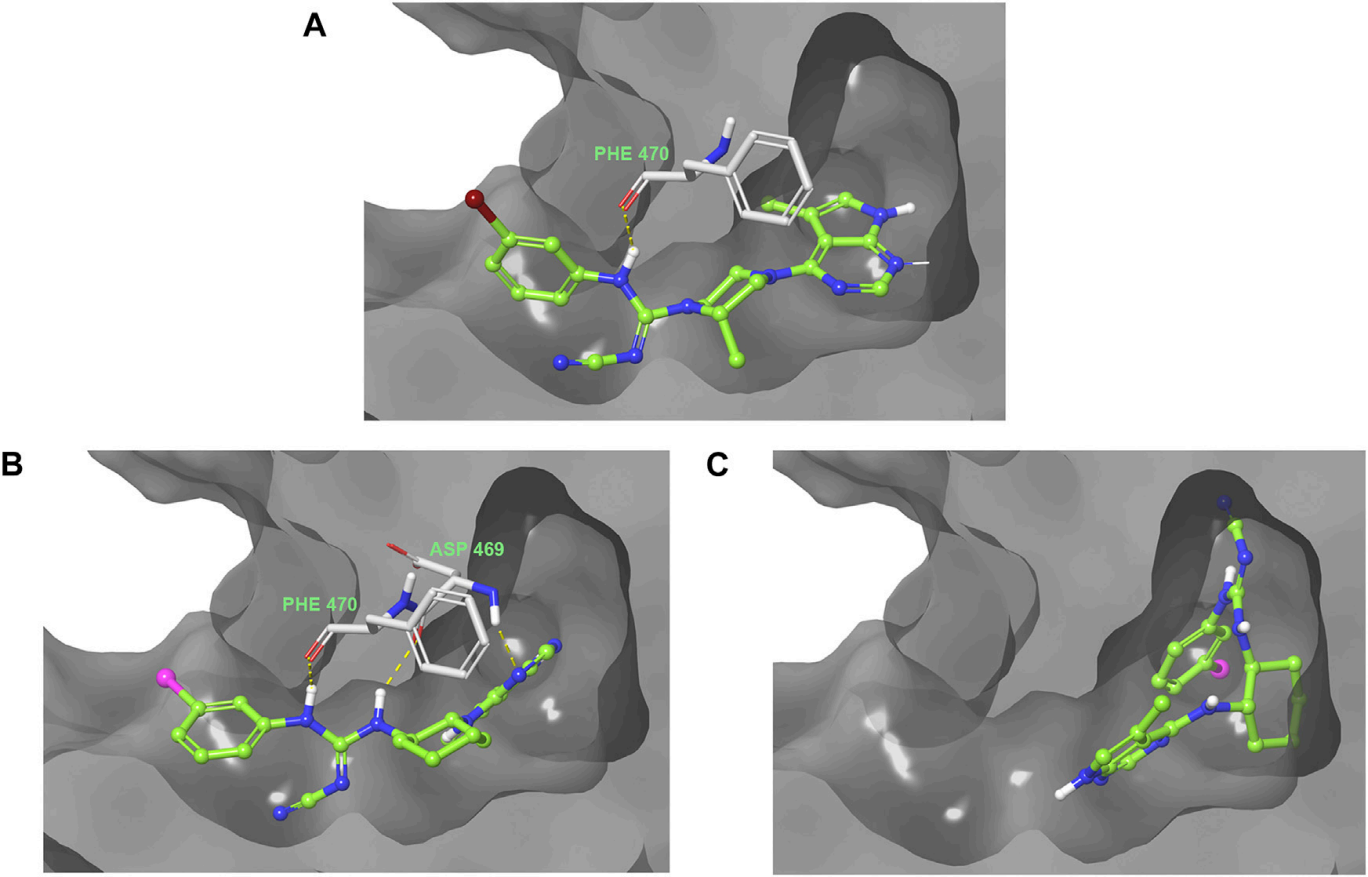
Predicted binding poses of ligand **1 (A)**, **24 (B)**, and **26 (C)** into the ATP-binding pocket of LIMK2.

The predicted binding pose toward LIMK2 is shown in Figure 5. Lead compound **1** formed a hydrogen bond between the cyanoguanidine moiety with Phe 470 of LIMK2. Although it only formed 1 hydrogen bond, the hydrophobic interaction with the surface residue could be accounted for its high potency. Compound **24** also formed hydrogen bonding with Phe 470, but the cyclohexyl moiety is directed differently compared to piperazine moiety in compound **1**. Due to this orientation, the hydrophobic interaction within the middle binding tunnel is different, which accounts for the different potency. Compound **26** bearing the (*R*, *R*)-cyclohexyl motif resulted in a binding pose with the iodophenyl moiety facing toward the inside of the binding pocket. This could be the reason for the lower inhibitory effect of **26** toward LIMK2.

Overall, changing the stereochemistry of the cyclohexyl group resulted in the switch of the binding pose. The orientation of the pyrrolopyrimidine moiety was detrimental to selectivity. When the pyrrolopyrimidine was oriented into the interior of one of the LIMK isoforms while facing outside on the other isoform, a binding selectivity was achieved.

## Conclusion

A series of new LIMK inhibitors have been synthesised based on lead compound **1**, in which the central linker portion is designed with a certain degree of flexibility. We identify the stereochemistry of 1,2-diaminocyclohexyl moiety as detrimental to LIMK isoform selectivity. The docking study revealed that these subtle stereochemistry differences alter the binding pose which reform the ligand-binding site interaction. We also investigated the effect of halogen substitution. We found that substitution of bromide to iodide in compound **22** improved the potency of lead compound **1**. Taken together, the selectivity achieved in this work will be valuable in aiding the development of more potent yet selective LIMK inhibitors for future studies.

## Materials and Methods

### Chemistry

All chemicals were purchased from a commercial source and used without any further purification. All HPLC purifications were performed in Shimadzu LC-20 AD equipped with a fraction collector. ^1^H-NMR and ^13^C-NMR were recorded on Bruker AVIIIHD 400 and 500 MHz. All melting points were measured in Stuart Digital SMP10. Mass spectrometry was measured on Shimadzu LC-MS 8050.

General synthetic procedure A: the mixture of pyrrolopyrimidine (1 mmol), diamino alkane (5 mmol), and DIPEA (3 mmol) in isopropanol (10 ml) was stirred at reflux overnight. Water was added to the reaction mixture, and the mixture was lyophilised. The crude product was purified with semi-preparative HPLC using the gradient of acetonitrile/water with 0.1% formic acid.

General synthetic procedure B: the mixture of 3-haloanoline (1 mmol) and diphenyl-N-cyanocarbonimidate (1 mmol) in acetonitrile (15 ml) was heated at 50°C overnight. The mixture was then cooled in ice bath, resulting in precipitation of the product. The crystalline solid was collected by filtration. The product was used for the next step without any further purification.

General synthetic procedure C: the amino or piperazine substituted pyrrolopyrimidine (1 mmol), phenyl carbaimidates (2 mmol), and Et_3_N (3 mmol) in acetonitrile (7 ml) and methanol (3 ml) was stirred at reflux overnight. Water was added to the reaction mixture, and the mixture was lyophilised. The crude product was purified with semi-preparative HPLC using gradient of acetonitrile/water with 0.1% formic acid.

(*S*)-Methyl piperazine **20**: the mixture of pyrrolopyrimidine **5** (1.2 mmol), (S)-tert-butyl 2-methylpiperazine-1-carboxylate (3.6 mmol), and DIPEA (3.6 mmol) in isopropanol (10 ml) was stirred at reflux overnight. The mixture was concentrated under vacuum. The residue was dissolved in 50% TFA in dichloromethane (10 ml) and stirred overnight at rt. The reaction was concentrated under vacuum, diluted with dichloromethane, and neutralised with sat. aq. sodium bicarbonate. The layers were separated, and the aqueous layer was back extracted with more dichloromethane. The combined organic layers were dried over MgSO_4_ and concentrated under vacuum. The crude product was purified with semi-preparative HPLC using the gradient of acetonitrile/water with 0.1% formic acid.

**1-(3-Chlorophenyl)-2-cyano-3-(2-((5-methyl-7*H*-pyrrolo[2,3-*d*]pyrimidin-4-yl)amino)ethyl)guanidine (8).** Total yield: 31%. Purity: >99%. mp: 87–88°C. ^1^H NMR (DMSO-d_6_, 400 MHz): δ 11.2 (s, 1H), 9.2 (s, 1H), 7.98–7.82 (m, 2H), 7.36–7.23 (m, 2H), 7.21–7.10 (m, 2H), 6.77 (m, 1H), 6.65 (m, 1H), 3.59 (m, 2H), 3.40 (m, 2H), 2.30 (d, *J* = 1.1 Hz, 3H); ^13^C NMR (DMSO-d_6_, 100 MHz): δ 163.5, 158.7, 157.1, 150.9, 139.9, 133.4, 130.8, 124.8, 123.6, 122.4, 119.4, 117.6, 109.0, 102.8, 42.6, 12.7; ESI-MS: [M + H] calc. 369.13, found 369.30. HR-MS (ESI+): [M + H] calc. 369.1343, found 369.1335.

**1-(3-Bromophenyl)-2-cyano-3-(2-((5-methyl-7*H*-pyrrolo[2,3-*d*]pyrimidin-4-yl)amino)ethyl)guanidine (9).** Total yield: 20%. Purity: >99%. mp: 86–88°C. ^1^H NMR (MeOD, 400 MHz): δ 9.36 (s, 1H), 8.08 (s, 1H), 7.39 (m, 1H), 7.31 (d, *J* = 7.8 Hz, 1H), 7.28 (m, 1H), 7.23–7.19 (m, 2H), 7.15–7.00 (m, 3H), 3.69 (t, *J* = 5.1 Hz, 2H), 3.55 (dd, *J* = 6.1, 4.4 Hz, 2H), 2.40 (d, *J* = 1.1 Hz, 3H); ^13^C NMR (MeOD, 125 MHz): δ 164.4, 159.2, 131.7, 130.6, 130.5, 130.1, 129.8, 129.0, 127.6, 127.5, 123.3, 120.5, 114.8, 113.5, 41.0, 41.0, 10.7; ESI-MS: [M + H] calc. 413.08, found 413.30. HR-MS (ESI+): [M + H] calc. 413.0838, found 413.0827.

**2-Cyano-1-(3-iodophenyl)-3-(2-((5-methyl-7*H*-pyrrolo[2,3-*d*]pyrimidin-4-yl)amino)ethyl)guanidine (10).** Total yield: 5%. Purity: >99%. mp: 88–89°C ^1^H NMR (MeOD, 400 MHz): δ 9.35 (s, 1H), 8.05 (s, 1H), 7.80 (t, *J* = 1.9 Hz, 1H), 7.61–7.56 (m, 2H), 7.48–7.37 (m, 3H), 7.05–6.99 (m, 2H), 3.67 (t, *J* = 6.0 Hz, 2H), 3.53 (dd, *J* = 4.1, 5.7 Hz, 2H), 2.39 (d, *J* = 1.1 Hz, 3H); ^13^C NMR (MeOD, 125 MHz): δ 164.4, 162.9, 159.2, 137.5, 136.6, 132.5, 130.6, 130.1, 129.9, 128.1, 123.7, 118.6, 114.3, 92.4, 46.6, 41.1, 10.7; ESI-MS: [M + H] calc. 461.06, found 461.25. HR-MS (ESI+): [M + H] calc. 461.0699, found 461.0689.

**1-(3-Chlorophenyl)-2-cyano-3-(3-((5-methyl-7*H*-pyrrolo[2,3-*d*]pyrimidin-4-yl)amino)propyl)guanidine (11).** Total yield: 7%. Purity: >99%. mp: 88–90°C. ^1^H NMR (MeOD, 400 MHz): δ 7.68 (s, 1H), 7.32–7.25 (m, 2H), 7.20–7.11 (m, 2H), 6.70 (d, *J* = 1.1 Hz, 1H), 3.53 (t, *J* = 6.2 Hz, 2H), 3.29 (t, *J* = 6.4 Hz, 2H), 2.35 (d, *J* = 1.1 Hz, 3H), 1.80–1.72 (m, 2H); ^13^C NMR (MeOD, 100 MHz): δ 158.8, 150.1, 138.4, 134.5, 130.4, 129.0, 125.9, 124.7, 122.9, 118.8, 117.5, 114.8, 109.8, 102.5, 38.3, 37.0, 29.5, 10.9; ESI-MS: [M + H] calc. 383.14, found 383.25. HR-MS (ESI+): [M + H] calc. 383.1499, found 383.1492.

**1-(3-Bromophenyl)-2-cyano-3-(3-((5-methyl-7*H*-pyrrolo[2,3-*d*]pyrimidin-4-yl)amino)propyl)guanidine 12).** Total yield: 30%. Purity: >99%. mp: 88–89°C. ^1^H NMR (MeOD, 400 MHz): δ 7.67 (s, 1H), 7.43 (m, 1H), 7.32 (m, 1H), 7.26–7.17 (m, 2H), 7.08–7.02 (m, 1H), 6.73–6.63 (m, 2H), 3.53 (t, *J* = 6.5 Hz, 2H), 3.29 (t, *J* = 6.5 Hz, 2H), 2.35 (d, *J* = 1.1 Hz, 3H), 1.76 (m, 2H); ^13^C NMR (MeOD, 100 MHz): δ 158.8, 157.2, 150.0, 149.1, 138.5, 130.6, 128.9, 127.6, 123.4, 118.9, 114.8, 109.8, 102.5, 38.3, 36.7, 29.5, 10.8; ESI-MS: [M + H] calc. 427.09, found 427.25. HR-MS (ESI+): [M + H] calc. 427.0994, found 427.0986.

**2-Cyano-1-(3-iodophenyl)-3-(3-((5-methyl-7*H*-pyrrolo[2,3-*d*]pyrimidin-4-yl)amino)propyl)guanidine (13).** Total yield: 17%. Purity: >99%. mp: 91–93°C. ^1^H NMR (MeOD, 400 MHz): δ 7.62 (t, *J* = 1.8 Hz, 2H), 7.52 (d, *J* = 7.9 Hz, 1H), 7.23 (m, 1H), 7.09–7.02 (m, 2H), 6.71–6.64 (m, 2H), 3.53 (t, *J* = 6.4 Hz, 2H), 3.28 (t, *J* = 6.4 Hz, 2H), 2.35 (d, *J* = 1.1 Hz, 3H), 1.75 (m, 2H); ^13^C NMR (MeOD, 100 MHz): δ 158.8, 157.3, 150.1, 149.1, 138.3, 135.0, 130.7, 129.0, 124.1, 118.8, 117.5, 114.8, 109.8, 102.5, 38.2, 37.0, 29.6, 10.9; ESI-MS: [M + H] calc. 475.07, found 475.30. HR-MS (ESI+): [M + H] calc. 475.0856, found 475.0847.

**1-(3-Chlorophenyl)-2-cyano-3-(4-((5-methyl-7*H*-pyrrolo[2,3-*d*]pyrimidin-4-yl)amino)butyl)guanidine (14).** Total yield: 55%. Purity: >99%. mp: 90–91°C. ^1^H NMR (MeOD, 400 MHz): δ 7.94 (s, 1H), 7.21 (t, *J* = 8.1 Hz, 1H), 7.19 (d, *J* = 2.1 Hz, 1H), 7.10 (m, 1H), 7.08–7.02 (m, 2H), 6.71 (d, *J* = 1.1 Hz, 1H), 6.67 (m, 1H), 3.48 (t, *J* = 6.4 Hz, 2H), 3.25 (t, *J* = 6.4 Hz, 2H), 2.33 (d, *J* = 1.1 Hz, 3H), 1.68–1.54 (m, 4H); ^13^C NMR (MeOD, 100 MHz): δ 158.6, 156.9, 150.0, 138.6, 134.3, 130.2, 128.9, 125.4, 124.1, 122.3, 118.9, 114.8, 109.8, 102.6, 41.3, 40.0, 26.5, 26.3, 10.8; ESI-MS: [M + H] calc. 397.16, found 397.25. HR-MS (ESI+): [M + H] calc. 397.1656, found 397.1650.

**1-(3-Bromophenyl)-2-cyano-3-(4-((5-methyl-7*H*-pyrrolo[2,3-*d*]pyrimidin-4-yl)amino)butyl)guanidine (15).** Total yield: 47%. Purity: >99%. mp: 97–98°C. ^1^H NMR (MeOD, 400 MHz): δ 7.94 (s, 1H), 7.35 (t, *J* = 1.8 Hz, 1H), 7.25 (m, 1H), 7.15 (t, *J* = 8.0 Hz, 1H), 7.10 (m, 1H), 7.05 (m, 1H), 6.72–6.63 (m, 2H), 3.48 (t, *J* = 6.9 Hz, 2H), 3.25 (t, *J* = 6.9 Hz, 2H), 2.33 (d, *J* = 1.1 Hz, 3H), 1.67–1.55 (m, 4H); ^13^C NMR (MeOD, 100 MHz): δ 158.6, 156.9, 149.9, 149.1, 138.7, 130.4, 129.0, 128.4, 127.0, 122.8, 118.9, 114.8, 109.8, 102.6, 41.3, 40.0, 26.5, 26.3, 10.8; ESI-MS: [M + H] calc. 441.11, found 441.30. HR-MS (ESI+): [M + H] calc. 441.1151, found 441.1141.

**2-Cyano-1-(3-iodophenyl)-3-(4-((5-methyl-7*H*-pyrrolo[2,3-*d*]pyrimidin-4-yl)amino)butyl)guanidine (16).** Total yield: 53%. Purity: >99%. mp: 90–91°C. ^1^H NMR (MeOD, 400 MHz): δ 7.94 (s, 1H), 7.53 (t, *J* = 1.7 Hz, 1H), 7.45 (m, 1H), 7.13 (m, 1H), 7.08–6.97 (m, 2H), 6.72–6.64 (m, 2H), 3.48 (t, *J* = 6.7 Hz, 2H), 3.24 (t, *J* = 6.9 Hz, 2H), 2.33 (d, *J* = 1.2 Hz, 3H), 1.67–1.54 (m, 4H); ^13^C NMR (MeOD, 125 MHz): δ 158.6, 156.9, 149.9, 138.4, 134.6, 133.0, 130.5, 129.0, 123.5, 118.9, 117.5, 114.8, 109.9, 102.6, 41.3, 40.0, 26.5, 26.3, 10.8; ESI-MS: [M + H] calc. 489.09, found 489.30. HR-MS (ESI+): [M + H] calc. 489.1012, found 489.1002.

**1-(3-Chlorophenyl)-2-cyano-3-((1*r*,4*r*)-4-((5-methyl-7*H*-pyrrolo[2,3-*d*]pyrimidin-4-yl)amino)cyclohexyl)guanidine (17).** Total yield: 3%. Purity: >99%. mp: 120–122 °C. ^1^H NMR (MeOD, 400 MHz): δ 7.94 (s, 1H), 7.29–7.19 (m, 2H), 7.14–7.06 (m, 2H), 6.71 (d, *J* = 1.2 Hz, 1H), 3.95 (m, 1H), 3.74 (m, 1H), 2.33 (d, *J* = 1.1 Hz, 3H), 2.09–1.93 (m, 4H), 1.50–1.37 (m, 4H); ^13^C NMR (MeOD, 125 MHz): δ 157.7, 156.6, 150.4, 149.5, 138.9, 134.4, 130.2, 125.1, 123.4, 121.7, 118.9, 117.3, 109.5, 102.7, 50.7, 48.6, 31.0, 30.8, 10.7; ESI-MS: [M + H] calc. 423.17, found 423.35. HR-MS (ESI+): [M + H] calc. 423.1812, found 423.1805.

**1-(3-Bromophenyl)-2-cyano-3-((1*r*,4*r*)-4-((5-methyl-7*H*-pyrrolo[2,3-*d*]pyrimidin-4-yl)amino)cyclohexyl)guanidine (18).** Total yield: 16%. Purity: >99%. mp: 127–128°C. ^1^H NMR (MeOD, 400 MHz): δ 7.94 (s, 1H), 7.36 (t, *J* = 1.9 Hz, 1H), 7.26 (m, 1H), 7.19 (t, *J* = 8.0 Hz, 1H), 7.13 (m, 1H), 6.72 (d, *J* = 1.2 Hz, 1H), 3.94 (m, 1H), 3.73 (m, 1H), 2.33 (d, *J* = 1.1 Hz, 3H), 2.09–1.92 (m, 4H), 1.51–1.37 (m, 4H); ^13^C NMR (MeOD, 100 MHz): δ 157.7, 156.5, 150.2, 149.4, 139.0, 130.4, 128.0, 126.3, 122.1, 122.1, 119.0, 117.3, 109.6, 102.6, 50.7, 48.7, 31.0, 30.8, 10.7; ESI-MS: [M + H] calc. 467.12, found 467.30. HR-MS (ESI+): [M + H] calc. 467.1307, found 467.1297.

**2-Cyano-1-(3-iodophenyl)-3-((1*r*,4*r*)-4-((5-methyl-7*H*-pyrrolo[2,3-*d*]pyrimidin-4-yl)amino)cyclohexyl)guanidine (19).** Total yield: 11%. Purity: >99%. mp: 129–130°C. ^1^H NMR (MeOD, 400 MHz): δ 9.36 (s, 1H), 8.02 (s, 1H), 7.59 (dt, *J* = 7.5, 1.5 Hz, 1H), 7.54 (t, *J* = 1.8 Hz, 1H), 7.46 (m, 1H), 7.38 (t, *J* = 2.0 Hz, 1H), 7.16 (m, 1H), 7.07–7.01 (m, 2H), 2.37 (d, *J* = 1.1 Hz, 3H), 2.02 (m, 4H), 1.64–1.36 (m, 4H); ^13^C NMR (MeOD, 125 MHz): δ 164.4, 157.7, 137.5, 136.5, 134.3, 132.4, 130.5, 129.9, 128.1, 122.8, 120.0, 117.3, 114.0, 93.4, 50.5, 49.4, 30.7, 30.7, 10.6; ESI-MS: [M + H] calc. 515.11, found 515.30. HR-MS (ESI+): [M + H] calc. 515.1169, found 515.1159.

**(*S*)-*N*′-Cyano-*N*-(3-iodophenyl)-2-methyl-4-(5-methyl-7*H*-pyrrolo[2,3-*d*]pyrimidin-4-yl)piperazine-1-carboximidamide (22).** Total yield: 7%. Purity: >99%. mp: 130–131°C. ^1^H NMR (MeOD, 400 MHz): δ 8.13 (s, 1H), 7.43–7.36 (m, 2H), 7.07–6.92 (m, 3H), 4.52 (m, 1H), 4.06 (m, 1H), 3.90 (m, 1H), 3.83 (m, 1H), 3.59 (m, 1H), 3.39 (dd, *J* = 13.1, 3.9 Hz, 1H), 3.09 (m, 1H), 2.36 (d, *J* = 1.1 Hz, 3H), 1.24 (d, *J* = 6.8 Hz, 3H); ^13^C NMR (MeOD, 125 MHz): δ 161.2, 158.4, 152.1, 149.4, 140.0, 133.1, 130.5, 130.1, 121.6, 120.5, 116.1, 109.1, 106.6, 93.4, 52.1, 50.8, 49.6, 41.4, 15.0, 12.5; ESI-MS: [M + H] calc. 501.09, found 501.20. HR-MS (ESI+): [M + H] calc. 501.1012, found 501.1003.

**1-(3-Bromophenyl)-2-cyano-3-((1*R*,2*R*)-2-((5-methyl-7*H*-pyrrolo[2,3-*d*]pyrimidin-4-yl)amino)cyclohexyl)guanidine (23).** Total yield: 8%. Purity: >99%. mp: 127–128°C. ^1^H NMR (MeOD, 400 MHz): δ 7.73 (s, 1H), 7.21 (m, 1H), 7.05 (t, *J* = 1.9 Hz, 1H), 7.00 (t, *J* = 8.0 Hz, 1H), 6.79 (m, 1H), 6.73 (d, *J* = 1.2 Hz, 1H), 4.05 (m, 1H), 3.84 (m, 1H), 2.37 (d, *J* = 1.2 Hz, 3H), 2.03 (m, 2H), 1.72 (m, 2H), 1.49–1.28 (m, 4H); ^13^C NMR (MeOD, 100 MHz): δ 158.5, 157.2, 150.4, 138.4, 136.2, 130.4, 128.6, 126.9, 122.6, 122.2, 119.1, 117.2, 109.6, 102.6, 56.3, 53.7, 32.1, 31.6, 24.5, 24.4, 10.9; ESI-MS: [M + H] calc. 467.12, found 467.30. HR-MS (ESI+): [M + H] calc. 467.1307, found 467.1297.

**2-Cyano-1-(3-iodophenyl)-3-((1*R*,2*R*)-2-((5-methyl-7*H*-pyrrolo[2,3-*d*]pyrimidin-4-yl)amino)cyclohexyl)guanidine (24).** Total yield: 9%. Purity: >99%. mp: 128–129°C. ^1^H NMR (MeOD, 400 MHz): δ 7.85 (s, 1H), 7.42 (td, *J* = 7.0, 1.8 Hz, 1H), 7.26 (m, 1H), 6.90–6.81 (m, 3H), 3.99 (m, 1H), 3.89 (m, 1H), 2.39 (d, *J* = 1.2 Hz, 2H), 2.03 (m, 2H), 1.72 (m, 2H), 1.52–1.28 (m, 4H); ^13^C NMR (MeOD, 125 MHz): δ 158.5, 147.9, 138.1, 134.8, 132.9, 130.5, 132.3, 120.0, 117.0, 110.5, 102.4, 93.5, 55.7, 54.6, 31.8, 31.5, 24.4, 24.3, 10.8; ESI-MS: [M + H] calc. 515.11, found 515.20. HR-MS (ESI+): [M + H] calc. 515.1169, found 515.1157.

**1-(3-Bromophenyl)-2-cyano-3-((1*S*,2*S*)-2-((5-methyl-7*H*-pyrrolo[2,3-*d*]pyrimidin-4-yl)amino)cyclohexyl)guanidine (25).** Total yield: 7%. Purity: >99%. mp: 127–128°C. ^1^H NMR (MeOD, 400 MHz): δ 7.72 (s, 1H), 7.21 (m, 1H), 7.05 (t, *J* = 1.9 Hz, 1H), 7.00 (t, *J* = 8.0 Hz, 1H), 6.79 (m, 1H), 6.73 (d, *J* = 1.2 Hz, 1H), 4.04 (m, 1H), 3.85 (m, 1H), 2.37 (d, *J* = 1.2 Hz, 3H), 2.03 (m, 2H), 1.71 (m, 2H), 1.48–1.27 (m, 4H); ^13^C NMR (MeOD, 100 MHz): δ 158.4, 157.1, 150.5, 149.6, 138.4, 130.5, 128.6, 126.9, 122.6, 122.2, 119.1, 117.2, 109.6, 102.6, 56.3, 53.7, 32.1, 31.6, 24.5, 24.4, 10.9; ESI-MS: [M + H] calc. 467.12, found 467.30. HR-MS (ESI+): [M + H] calc. 467.1307, found 467.1297.

**2-Cyano-1-(3-iodophenyl)-3-((1*S*,2*S*)-2-((5-methyl-7*H*-pyrrolo[2,3-*d*]pyrimidin-4-yl)amino)cyclohexyl)guanidine (26).** Total yield: 11%. Purity: >99%. mp: 128–129°C. ^1^H NMR (MeOD, 400 MHz): δ 7.74 (s, 1H), 7.42 (td, *J* = 7.3, 1.6 Hz, 1H), 7.26 (t, *J* = 2.0 Hz, 1H), 6.88–6.79 (m, 2H), 6.74 (d, *J* = 1.1 Hz, 1H), 4.03 (m, 1H), 3.85 (m, 1H), 2.37 (d, *J* = 1.2 Hz, 2H), 2.03 (m, 2H), 1.71 (m, 2H), 1.49–1.27 (m, 4H); ^13^C NMR (MeOD, 100 MHz): δ 158.5, 157.1, 150.4, 149.7, 138.2, 134.7, 132.9, 130.5, 123.3, 119.1, 117.2, 109.6, 102.6, 93.5, 56.2, 53.8, 32.1, 31.6, 24.5, 24.4, 10.9; ESI-MS: [M + H] calc. 515.11, found 515.25. HR-MS (ESI+): [M + H] calc. 515.1169, found 515.1157.

### *In Silico* Docking Study

The molecular docking study of all compounds was carried out in Schrodinger Glide Software package. The 2D structures of the newly synthetic compounds were accurately drawn using ChemDraw 19.0 software and then exported to Schrodinger Maestro. Crystal structures were prepared by downloading the pdf file from the Protein Data Bank (LIMK1: 5NXC; LIMK2: 4TPT). Protein was prepared using protein preparation wizard within the Schrodinger package using default settings. Receptor grids are set up as 10 Å radius around the ATP binding pocket of LIMK. Inhibitors were docked into the receptor using the Glide SP method, and the pose with the lowest GLIDE binding score was selected for study. Schrodinger Maestro 12.7 was then used to visualise the interactions between the ligand molecules and LIMK.

### Biological Study

#### LIMK1 ADP-Glo™ Kinase Assay System

ADP-Glo kinase assay was performed in 96-well plate format using the Promega kinase assay system. LIMK1 (0.1 μg) and compounds (0.1–100 μM) were dissolved in kinase reaction buffer and incubated for 30 min at rt. The reaction was then initiated by the addition of ATP (10 μM, 5 μl) and incubation for 30 min at 30°C. The reaction was then quenched with addition of 25 μl of ADP-Glo reagent. Kinase detection reagent (50 μl) was added and incubated for 30 min prior to luminescence reading on the BMG Labtech PHERAstar FS plate reader. Kinase assay is performed as duplicate.

#### ^33^P-ATP Radiotracer Assay

The radiotracer assay of LIMK1 and LIMK2 was performed by Reaction Biology Corporation. Compounds were tested in 10-dose IC_50_ mode with fivefold series dilution starting at 10 μM. Reactions were carried out at 10 μM ^33^P-ATP, 1 μM cofilin substrate, and 50 nM LIMK1 (final concentration). Kinase activity is determined by the transfer or radioactive ^33^P from ATP to cofilin substrate. Radiotracer assay is performed as triplicate.

#### Scratch Assay

The velocity of cell migration is measured using the C6 cell line in the 24-well plate. Cells were seeded at 100,000 cells/well. After 24 h, cells were washed with warm PBS and treated with the inhibitors. Directly following the treatment, cells were moved into a preheated stage-top incubator maintained at 37°C/5% CO_2_, using the ZEISS Axio Observer live cell microscope, fitted with an Axiocam 702 mono camera. Cells were imaged once every 10 min using a ×10 objective with phase contrast for a 24-h period. Cell migration was analysed using Fiji ImageJ (NIH) and Chemotaxis Migration Tool (ibidi).

## Data Availability

The original contributions presented in the study are included in the article/Supplementary Material, further inquiries can be directed to the corresponding author.
