# Supplementary material for: The Nature of Diamino Linker and Halogen Bonding Define Selectivity of Pyrrolopyrimidine-Based LIMK1 Inhibitors
Source: Front Chem. 2021 Dec 13;9:781213. doi: 10.3389/fchem.2021.781213 (PMC8711653; doi:10.3389/fchem.2021.781213)
Supplement: Supplementary file 4 [file Table1.DOCX]

Supplementary Information

**The nature of diamino linker and halogen bonding define selectivity of pyrrolopyrimidine based LIMK1 inhibitors.**

Daryl Ariawan^1^, Carol Au^1^, Esmeralda Paric^1^, Thomas Fath^1^, Yazi D. Ke^1^, Michael Kassiou^2^, Janet van Eersel^1^, Lars M. Ittner^1*^

^1^Dementia Research Centre, Department of Biomedical Science, Faculty of Medicine and Health Sciences, Macquarie University, Sydney, NSW 2109, Australia.

^2^ School of Chemistry, The University of Sydney, NSW 2006, Australia

Contents:

Spectra of novel compounds……………………………………………………………………………………………………………...2

**Fig. S1** – Comparison of LIMK1 crystal structure of 5NXC ligand vs predicted binding pose………………..36

**Fig. S2** – Comparison of LIMK2 crystal structure of 4TPT ligand vs predicted binding pose…………………36

**Table S1** – Docking energy of compound **1, 24** and **26**……………………………………………………………………….37

**1-(3-chlorophenyl)-2-cyano-3-(2-((5-methyl-7*H*-pyrrolo[2,3-*d*]pyrimidin-4-yl)amino)ethyl)guanidine (8).**

^1^H NMR (DMSO-d_6_, 400 MHz)
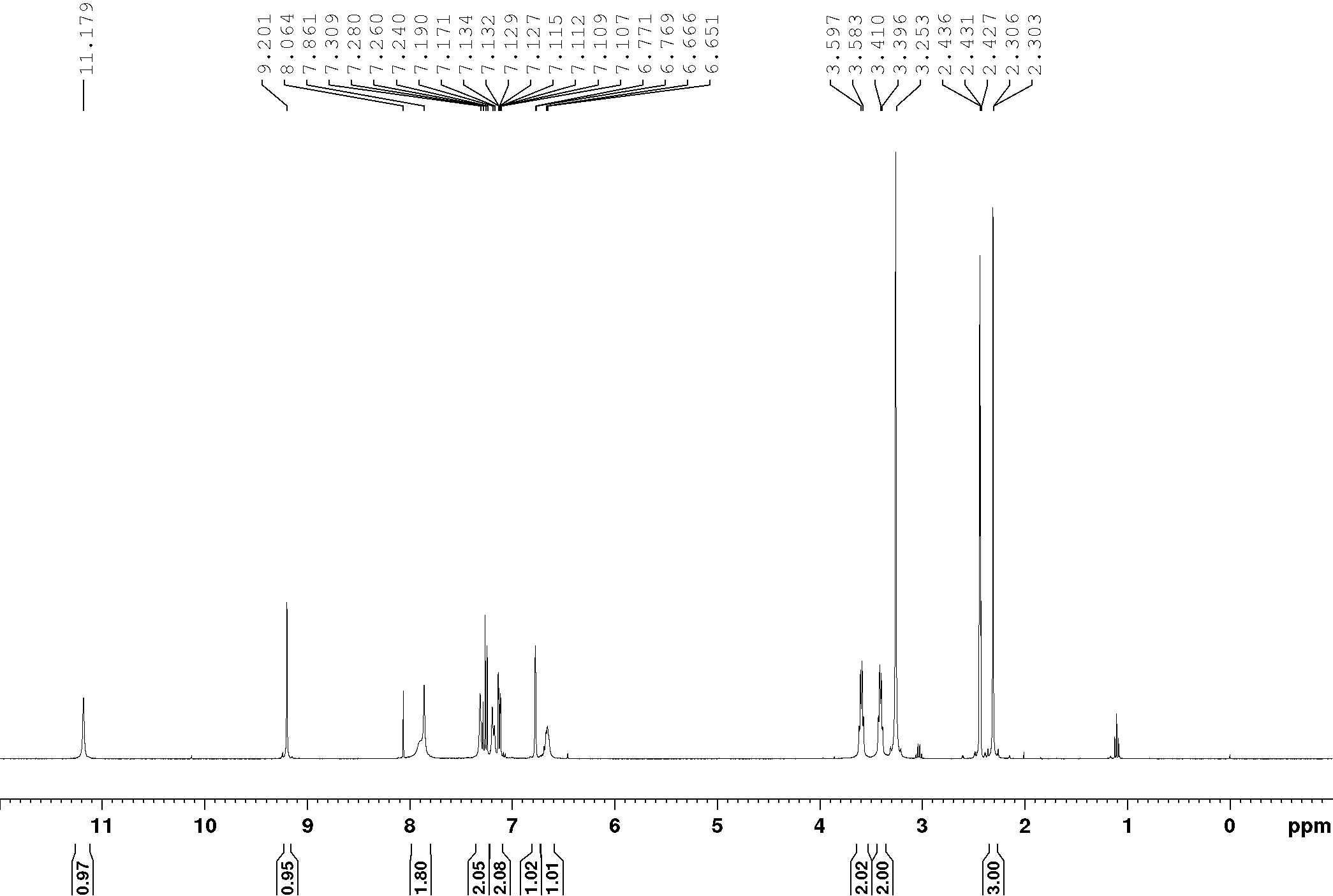


^13^C NMR (DMSO-d_6_, 100 MHz)


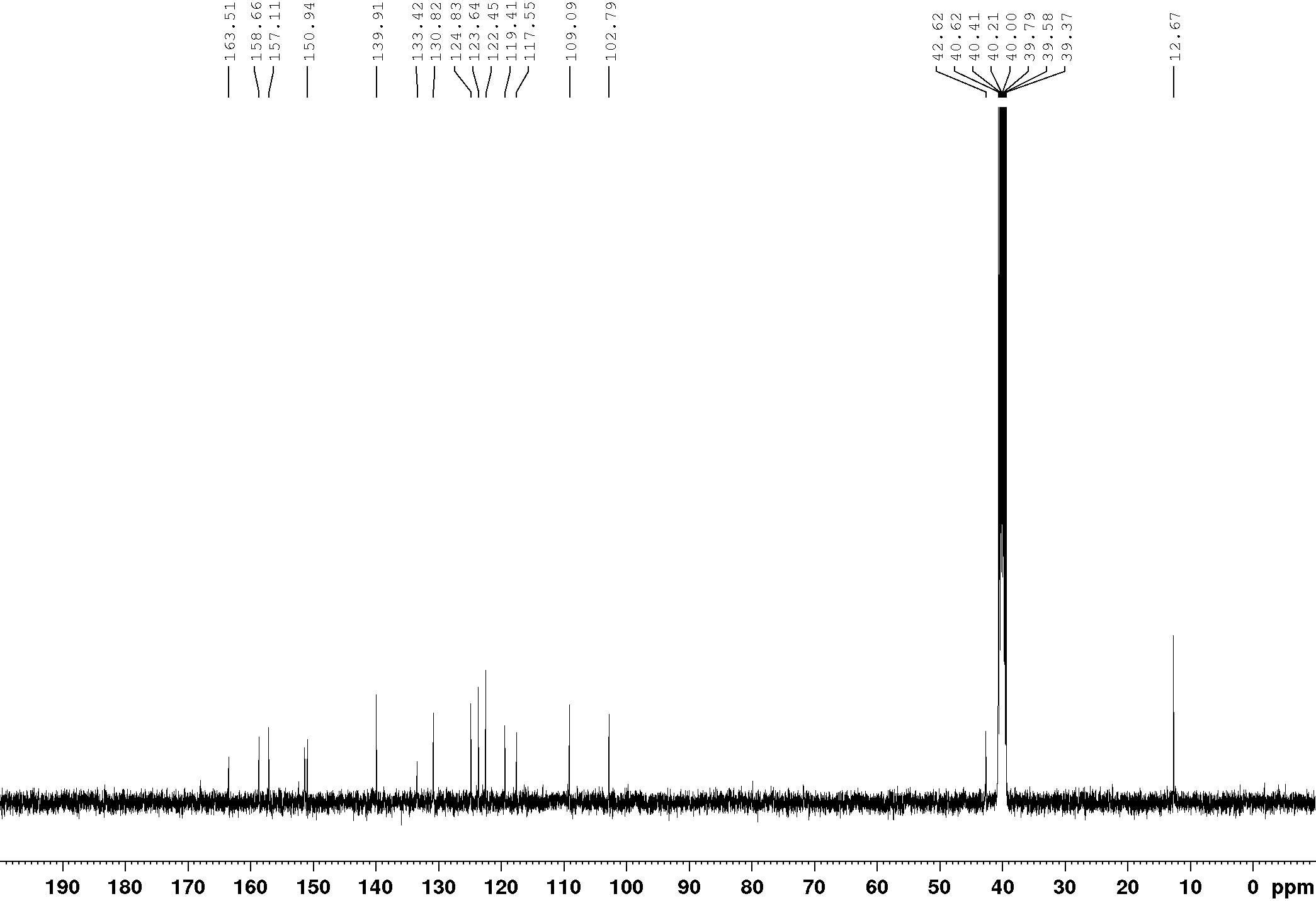


Analytical HPLC trace

HRMS ESI+ spectra

**1-(3-bromophenyl)-2-cyano-3-(2-((5-methyl-7*H*-pyrrolo[2,3-*d*]pyrimidin-4-yl)amino)ethyl)guanidine (9).**

^1^H NMR (MeOD, 400 MHz)


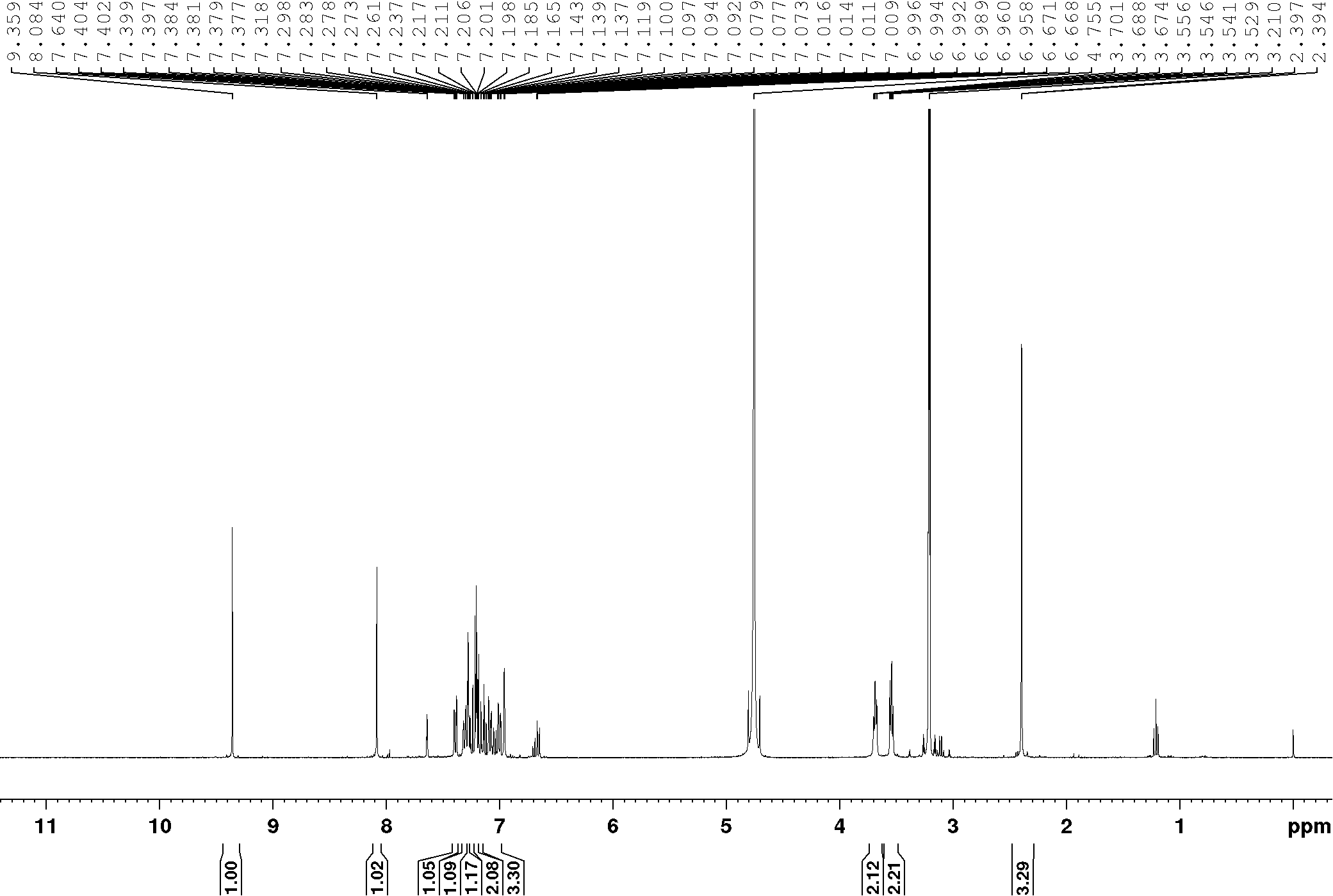


^13^C NMR (MeOD, 125 MHz)


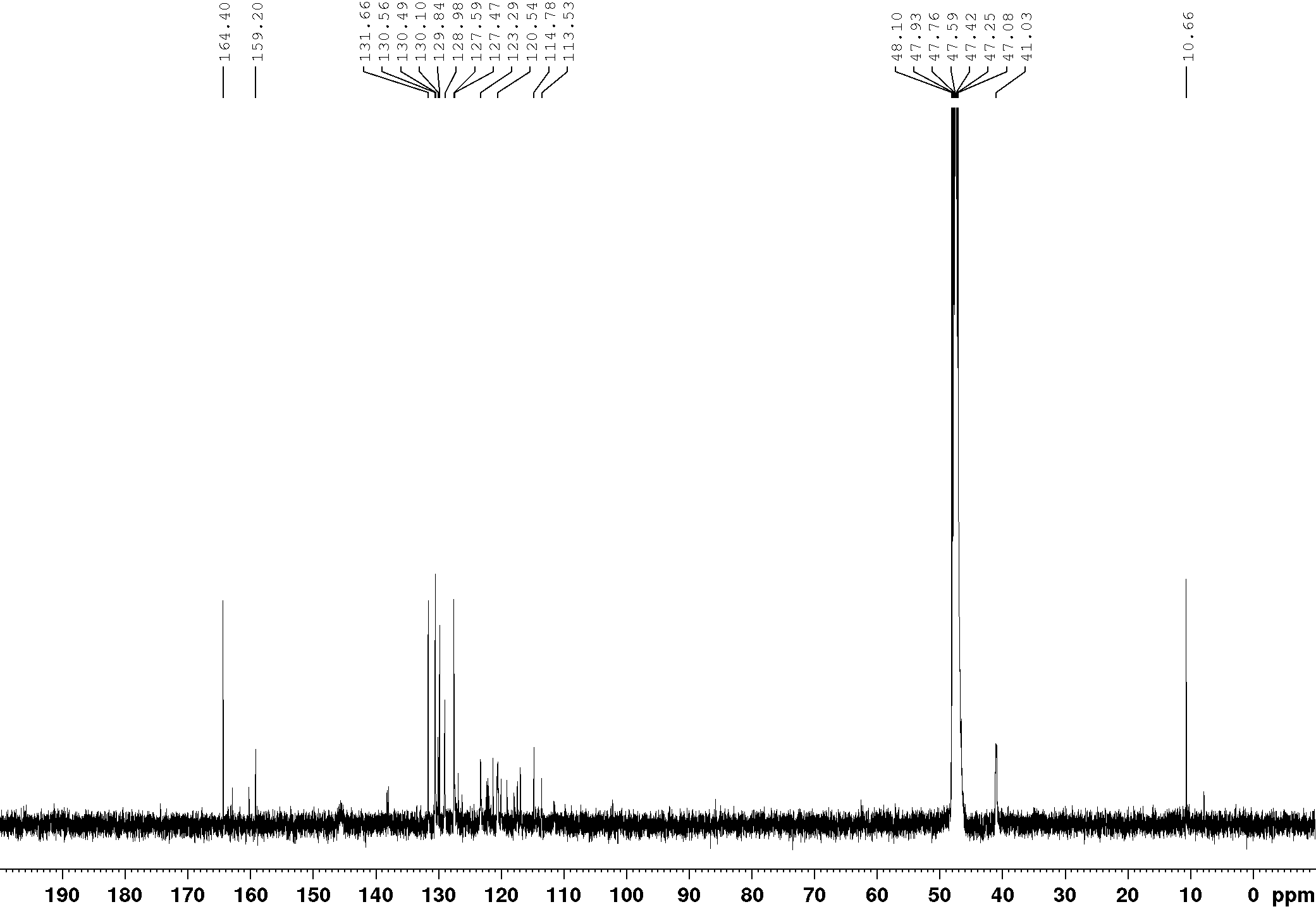


Analytical HPLC trace

HRMS ESI+ spectra

**2-cyano-1-(3-iodophenyl)-3-(2-((5-methyl-7*H*-pyrrolo[2,3-*d*]pyrimidin-4-yl)amino)ethyl)guanidine (10).**

^1^H NMR (MeOD, 400 MHz)


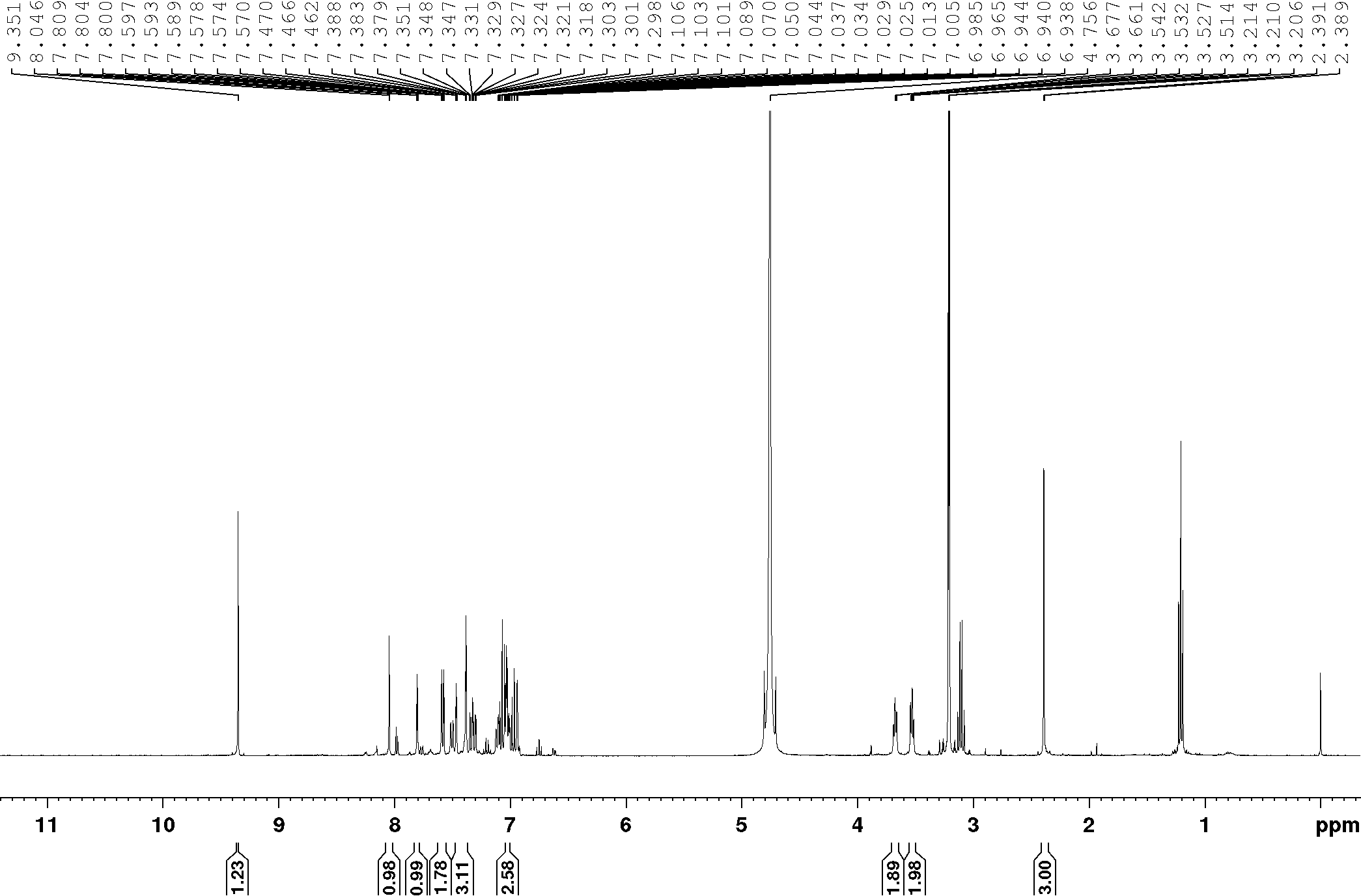


^13^C NMR (MeOD, 100 MHz)


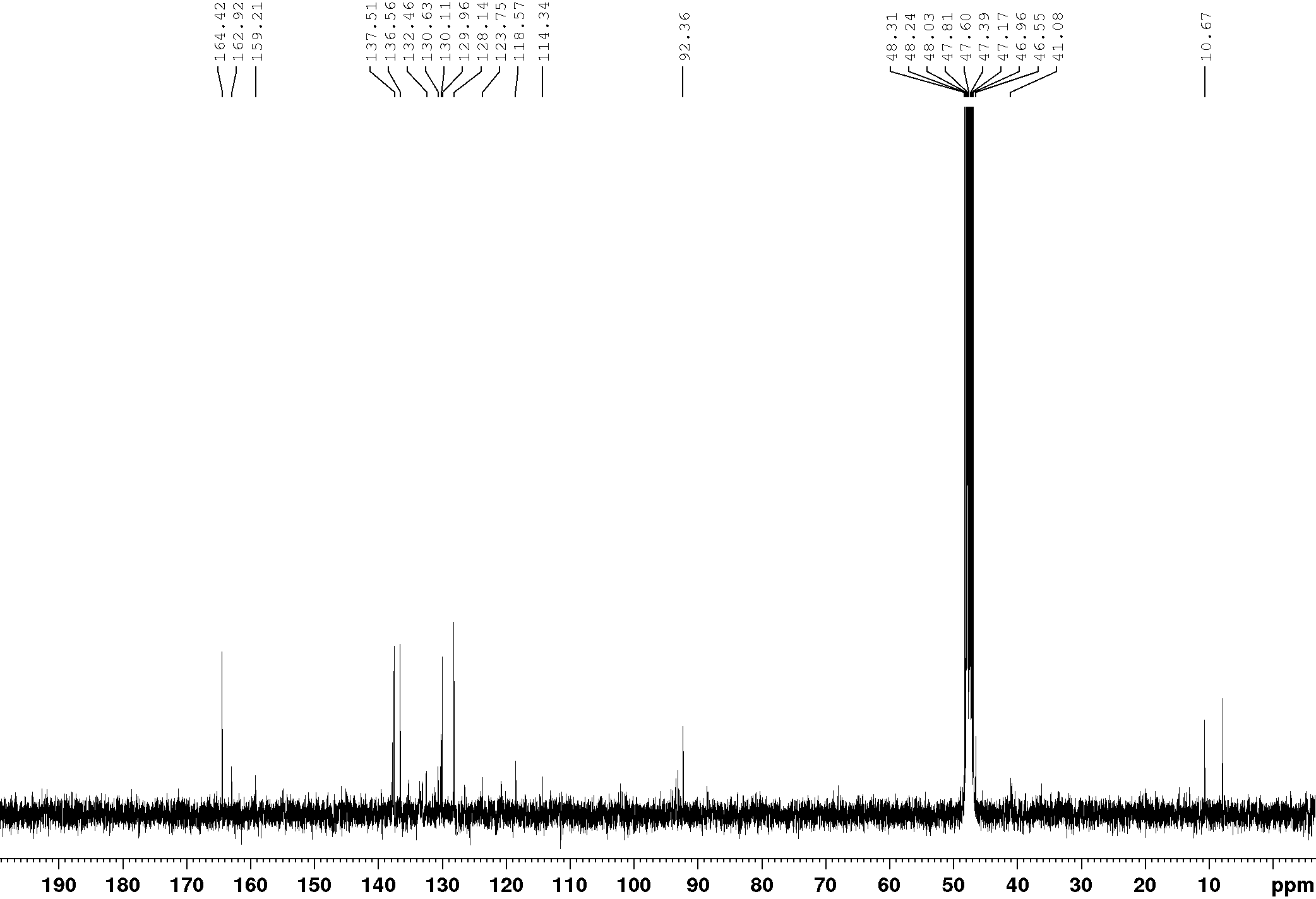


Analytical HPLC trace

HRMS ESI+ spectra

**1-(3-chlorophenyl)-2-cyano-3-(3-((5-methyl-7*H*-pyrrolo[2,3-*d*]pyrimidin-4-yl)amino)propyl)guanidine (11).**

^1^H NMR (MeOD, 400 MHz)


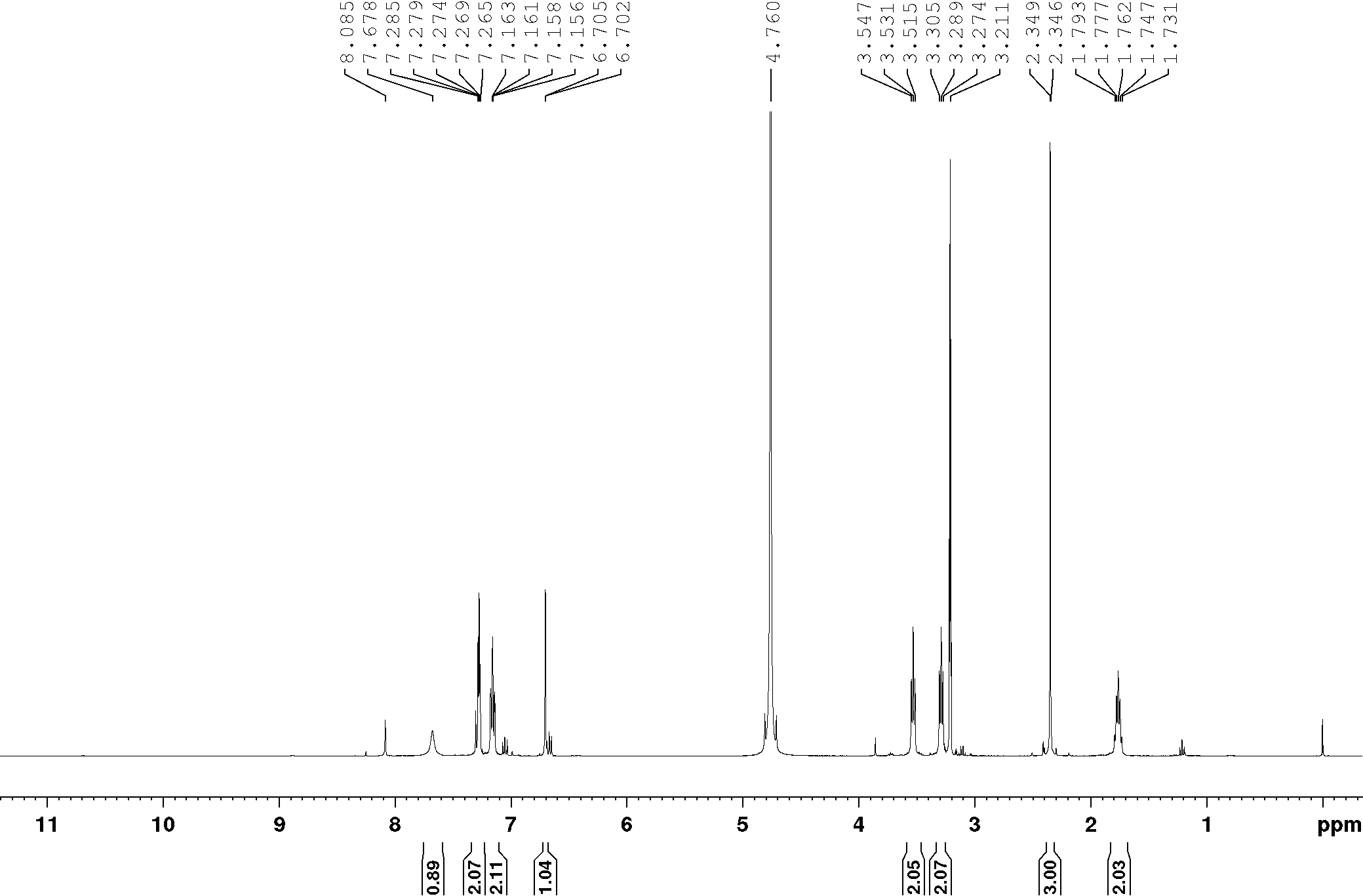


^13^C NMR (MeOD, 100 MHz)


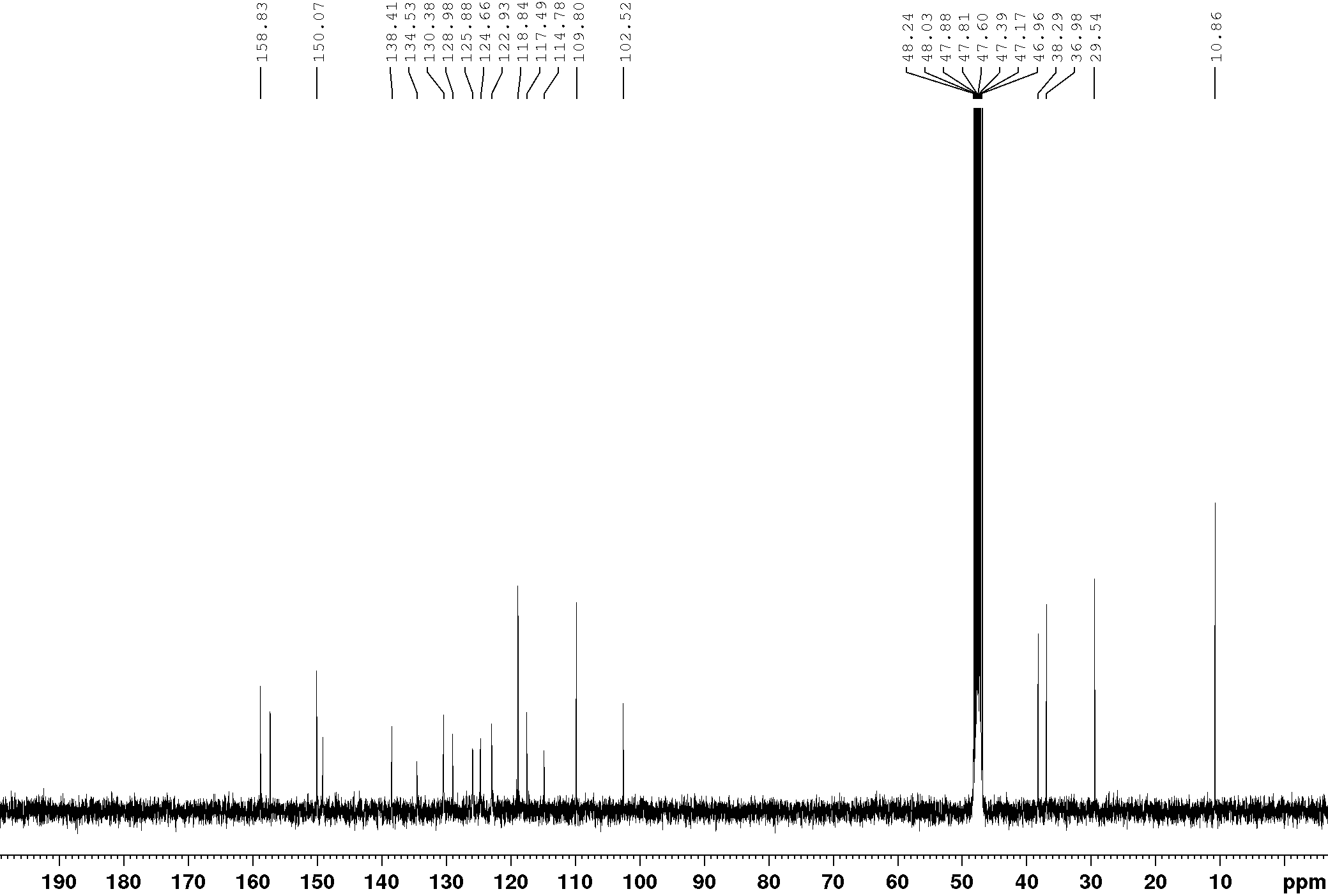


Analytical HPLC trace

HRMS ESI+ spectra

**1-(3-bromophenyl)-2-cyano-3-(3-((5-methyl-7*H*-pyrrolo[2,3-*d*]pyrimidin-4-yl)amino)propyl)guanidine (12).**

^1^H NMR (MeOD, 400 MHz)


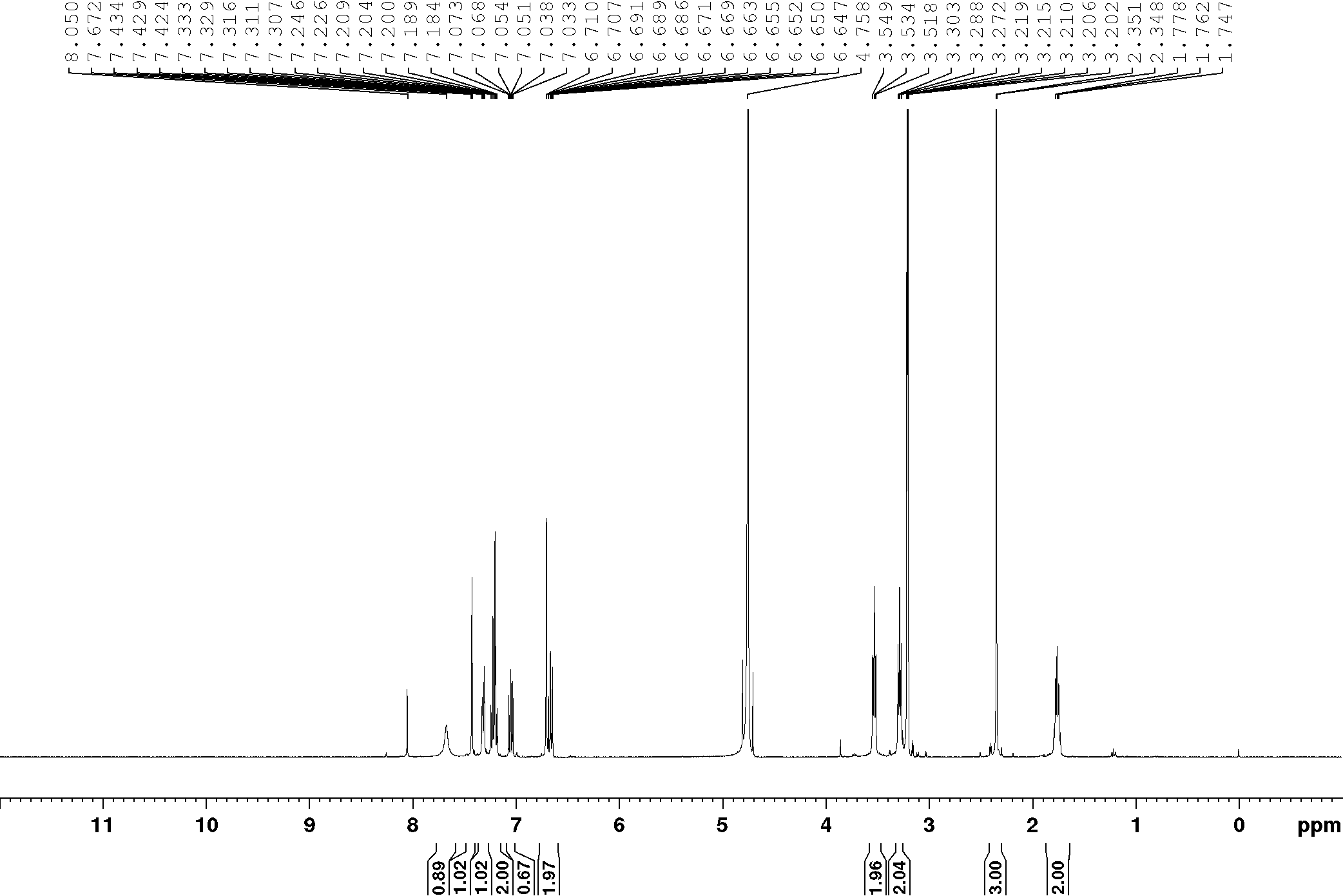


^13^C NMR (MeOD, 100 MHz)


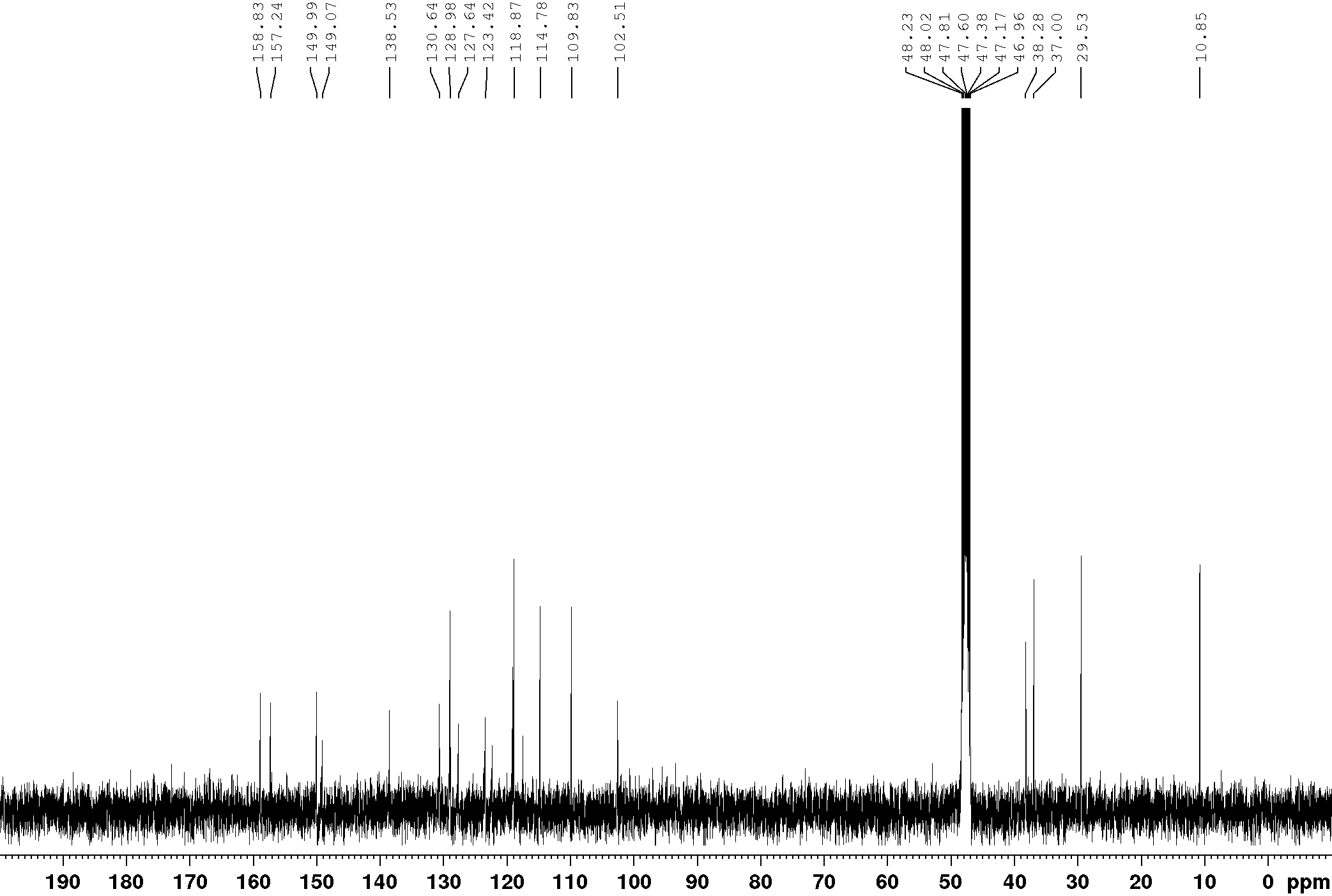


Analytical HPLC trace

HRMS ESI+ spectra

**2-cyano-1-(3-iodophenyl)-3-(3-((5-methyl-7*H*-pyrrolo[2,3-*d*]pyrimidin-4-yl)amino)propyl)guanidine (13).**

^1^H NMR (MeOD, 400 MHz)


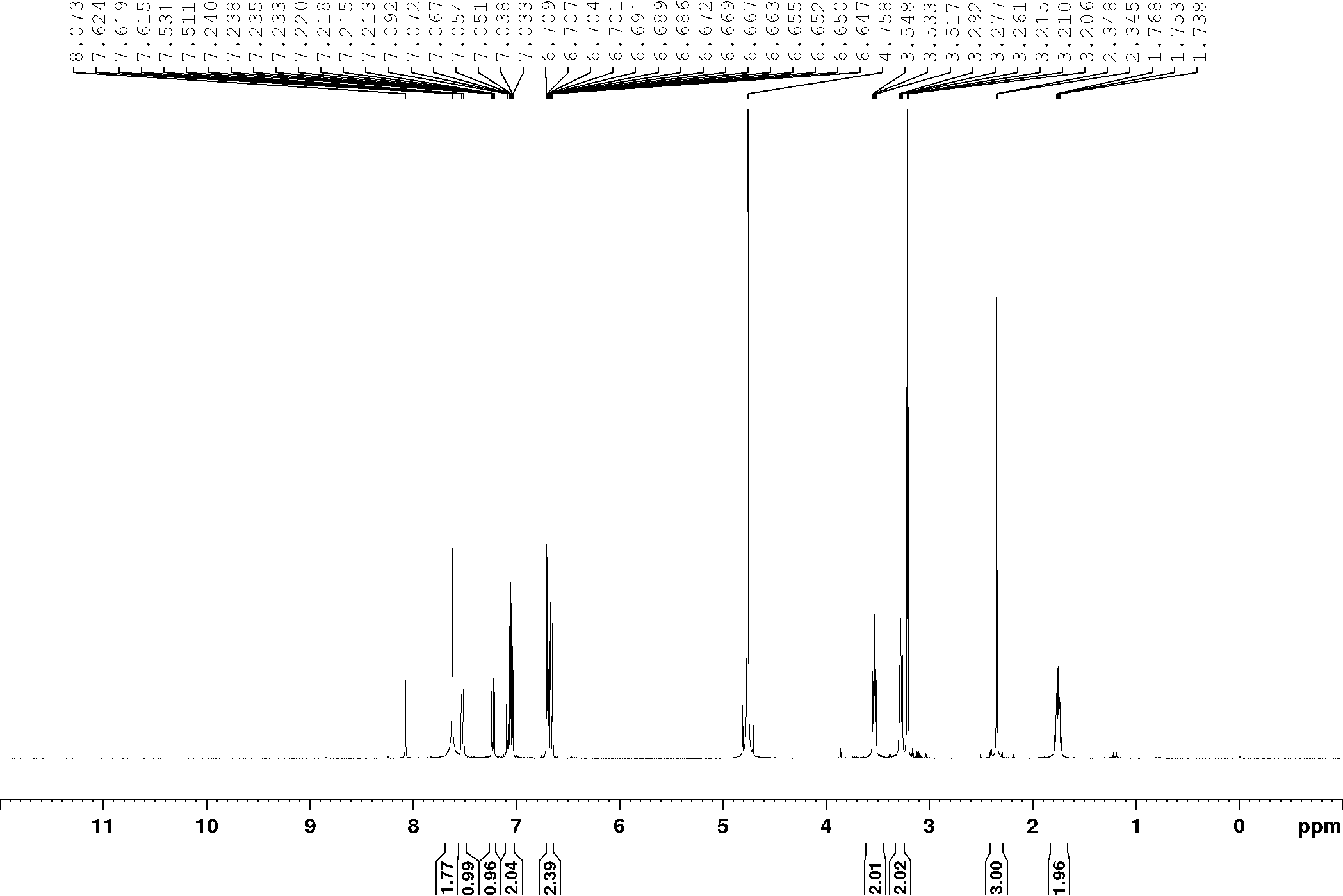


^13^C NMR (MeOD, 100 MHz)


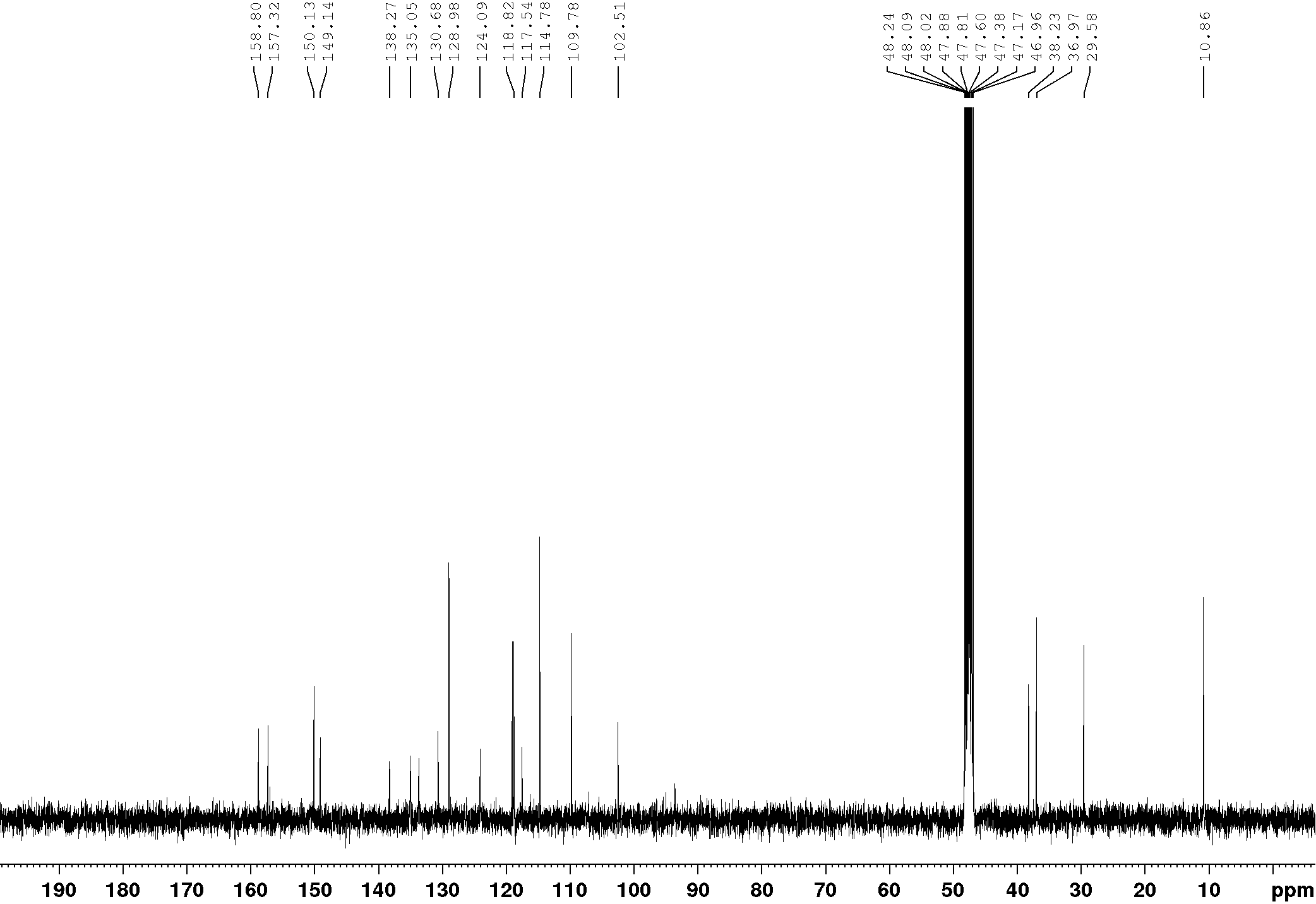


Analytical HPLC trace

HRMS ESI+ spectra

**1-(3-chlorophenyl)-2-cyano-3-(4-((5-methyl-7*H*-pyrrolo[2,3-*d*]pyrimidin-4-yl)amino)butyl)guanidine (14).**

^1^H NMR (MeOD, 400 MHz)


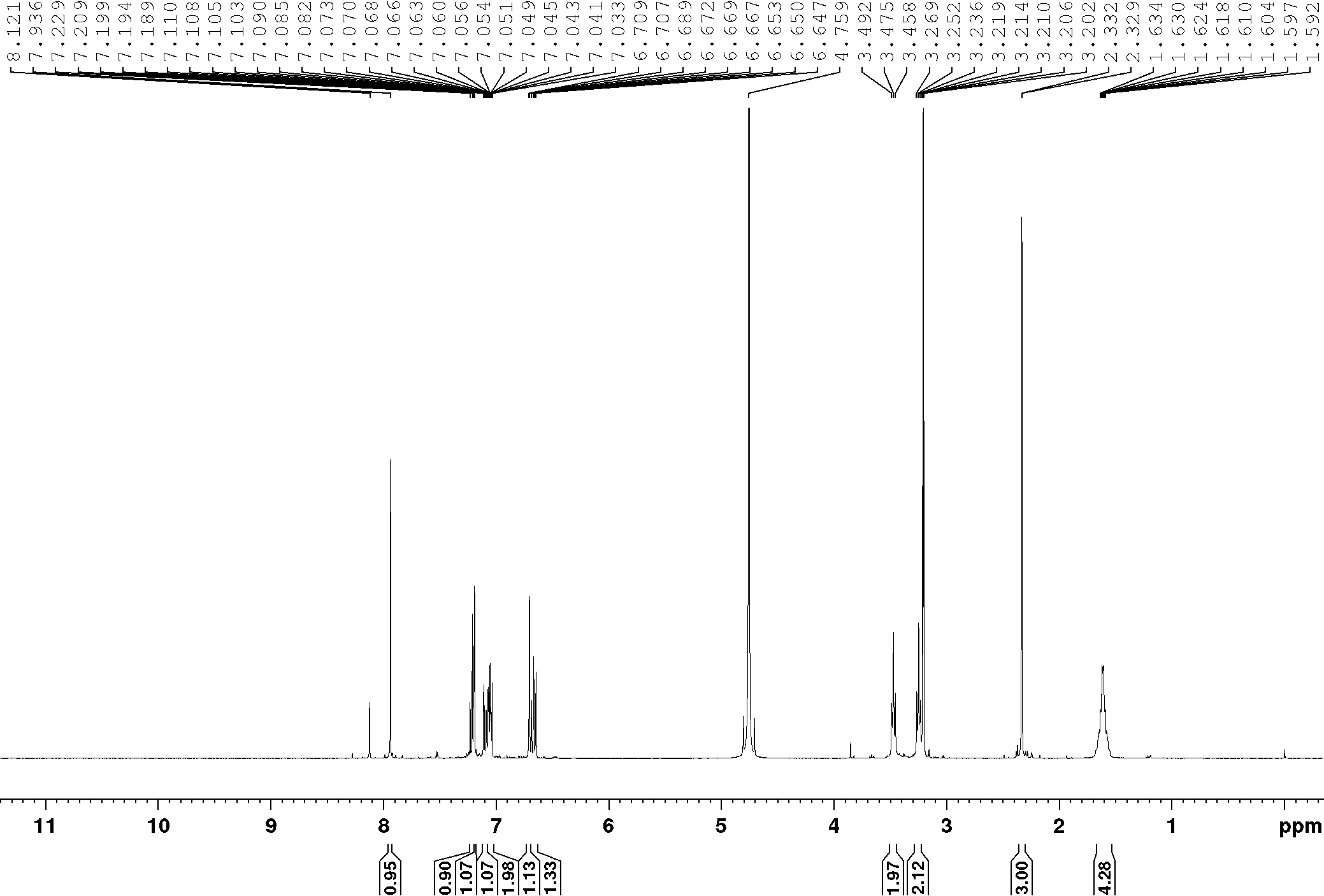


^13^C NMR (MeOD, 100 MHz)


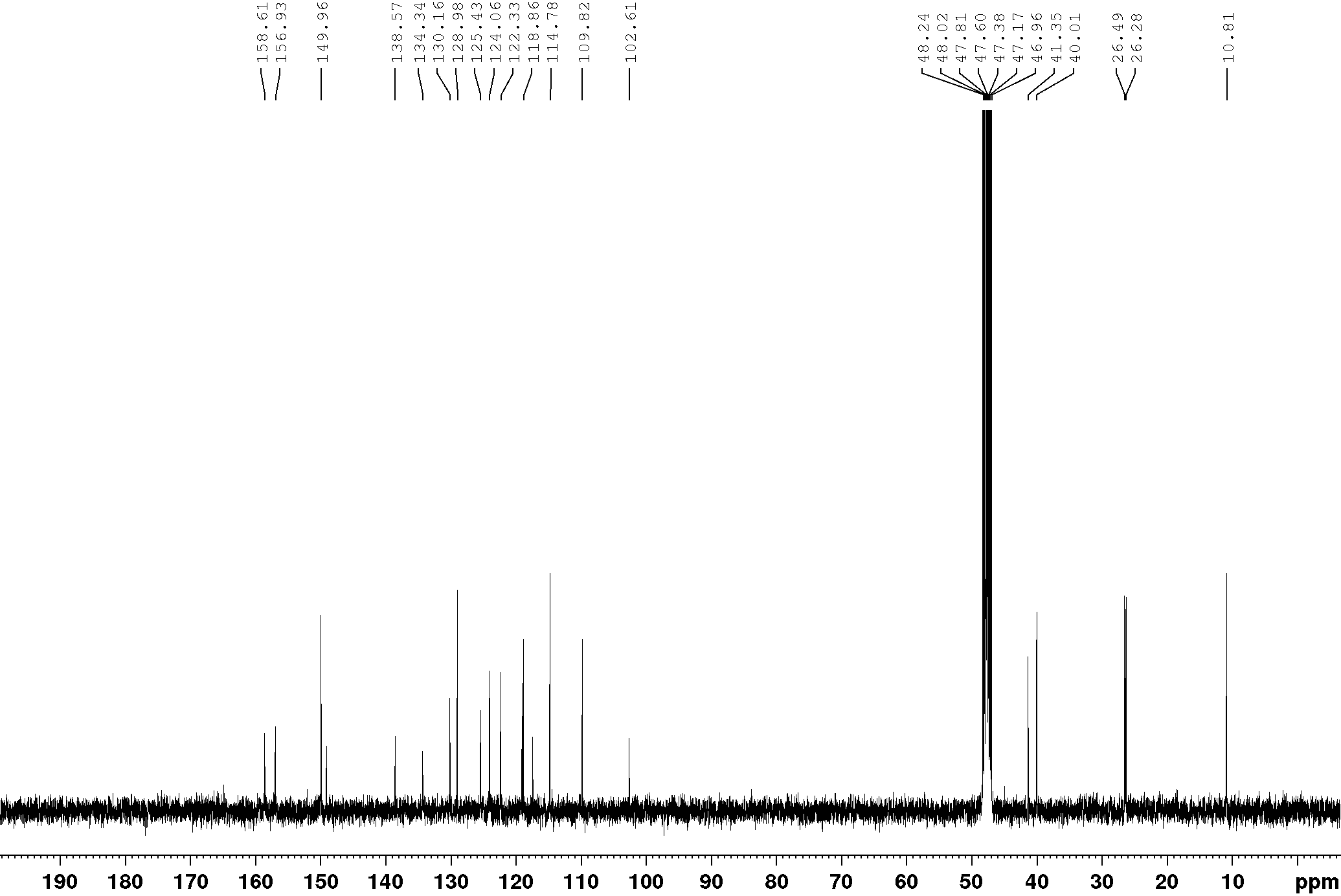


Analytical HPLC trace

HRMS ESI+ spectra

**1-(3-bromophenyl)-2-cyano-3-(4-((5-methyl-7*H*-pyrrolo[2,3-*d*]pyrimidin-4-yl)amino)butyl)guanidine (15).**

^1^H NMR (MeOD, 400 MHz)


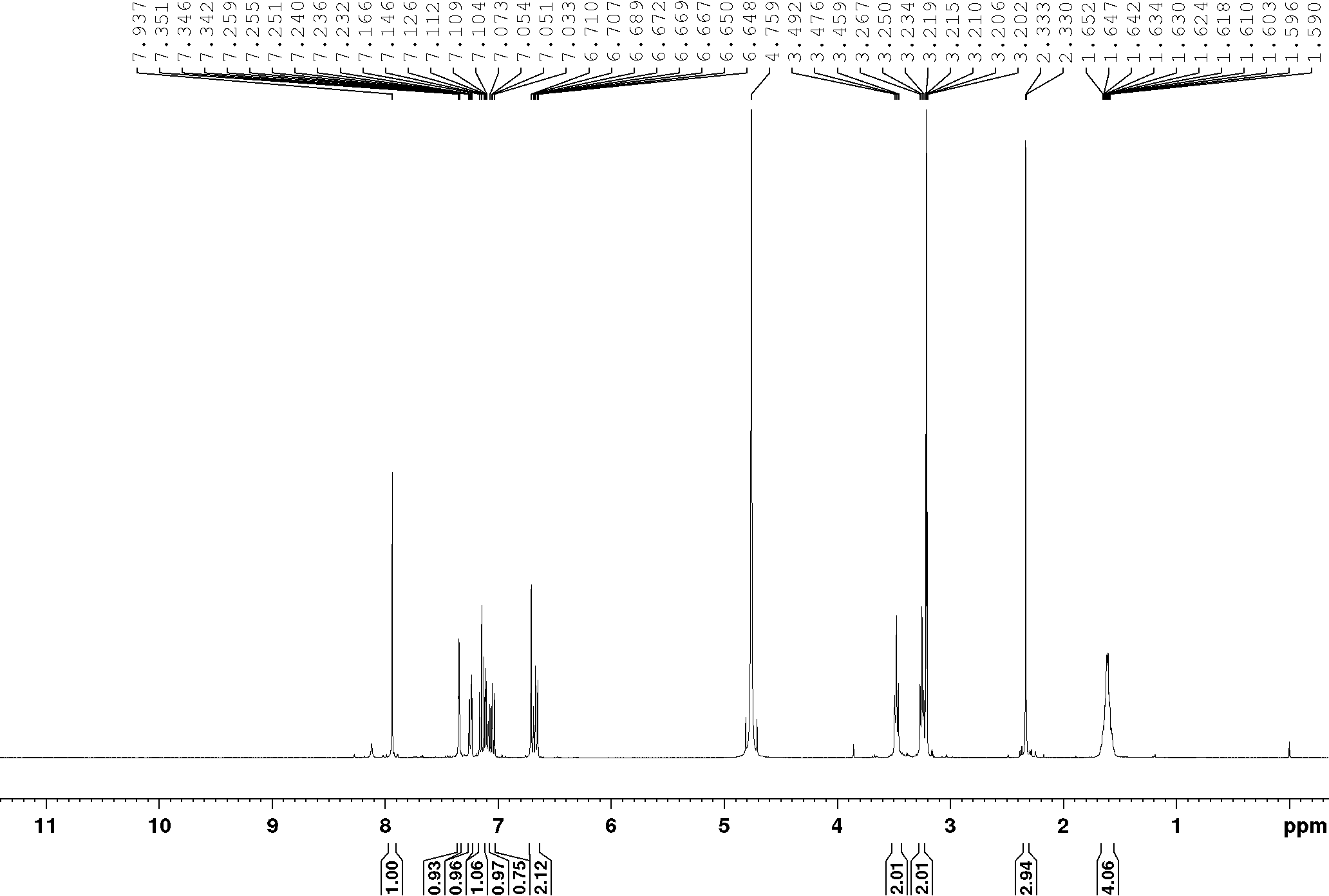


^13^C NMR (MeOD, 100 MHz)


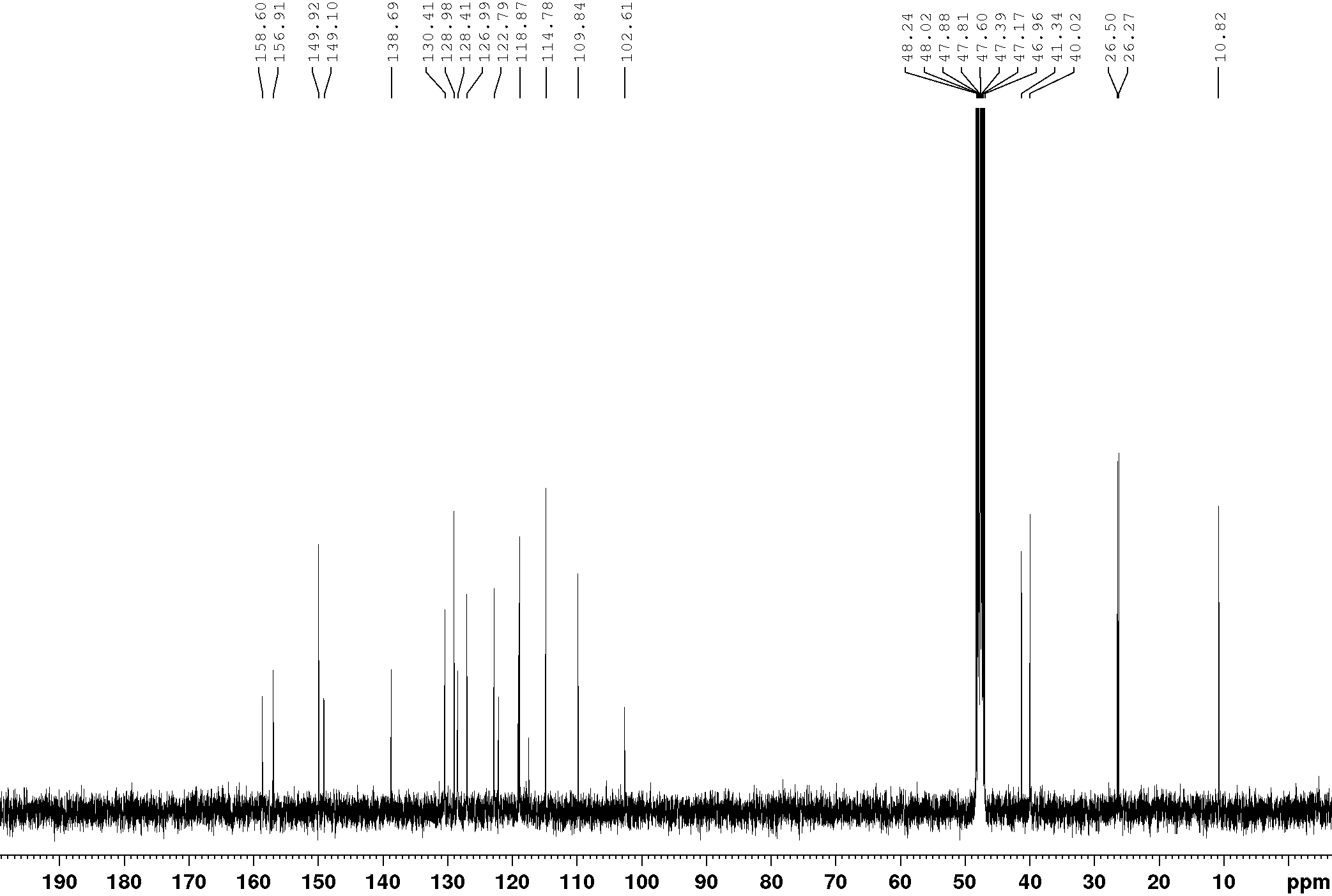


Analytical HPLC trace

HRMS ESI+ spectra

**2-cyano-1-(3-iodophenyl)-3-(4-((5-methyl-7*H*-pyrrolo[2,3-*d*]pyrimidin-4-yl)amino)butyl)guanidine (16).**

^1^H NMR (MeOD, 400 MHz)


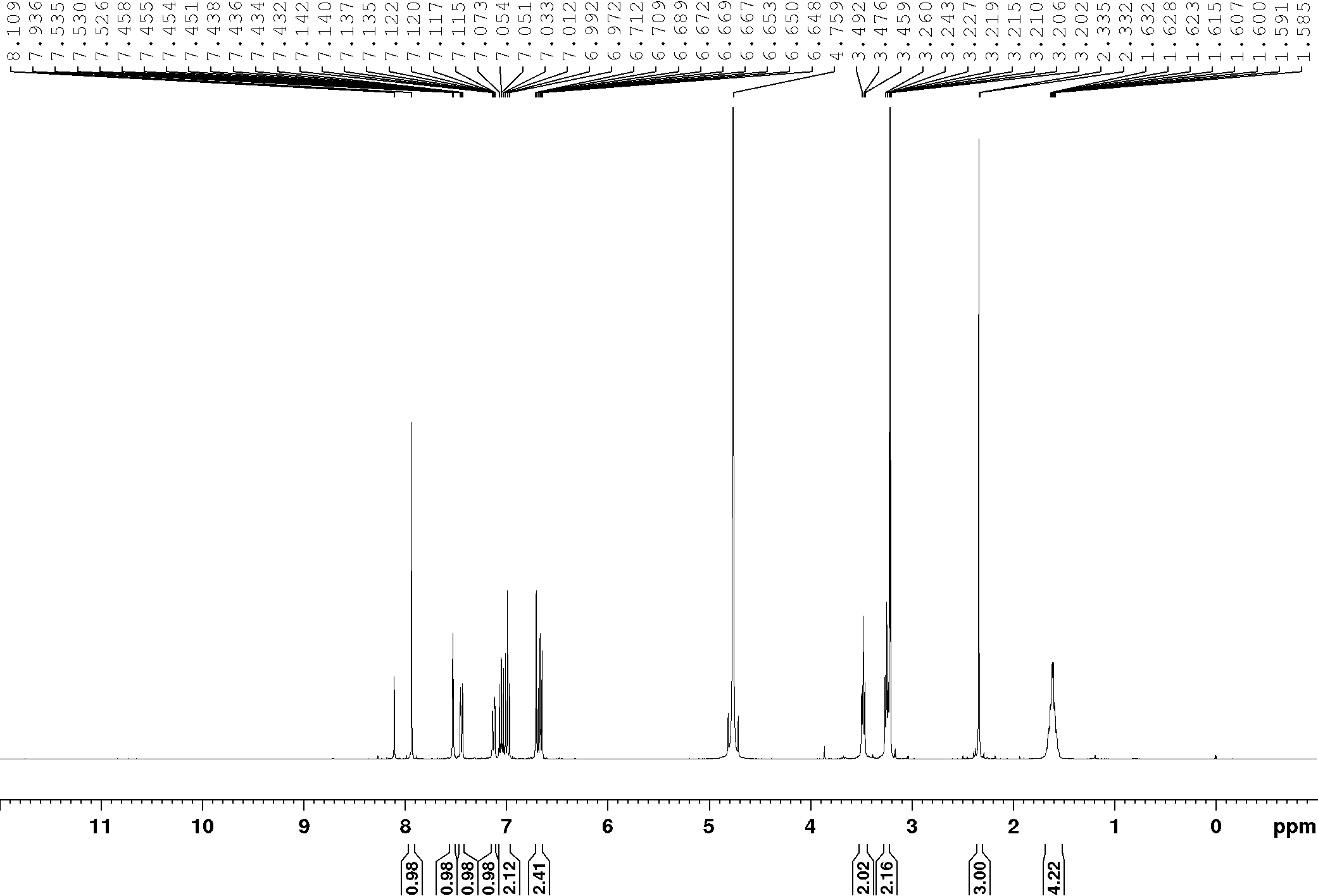


^13^C NMR (MeOD, 125 MHz)


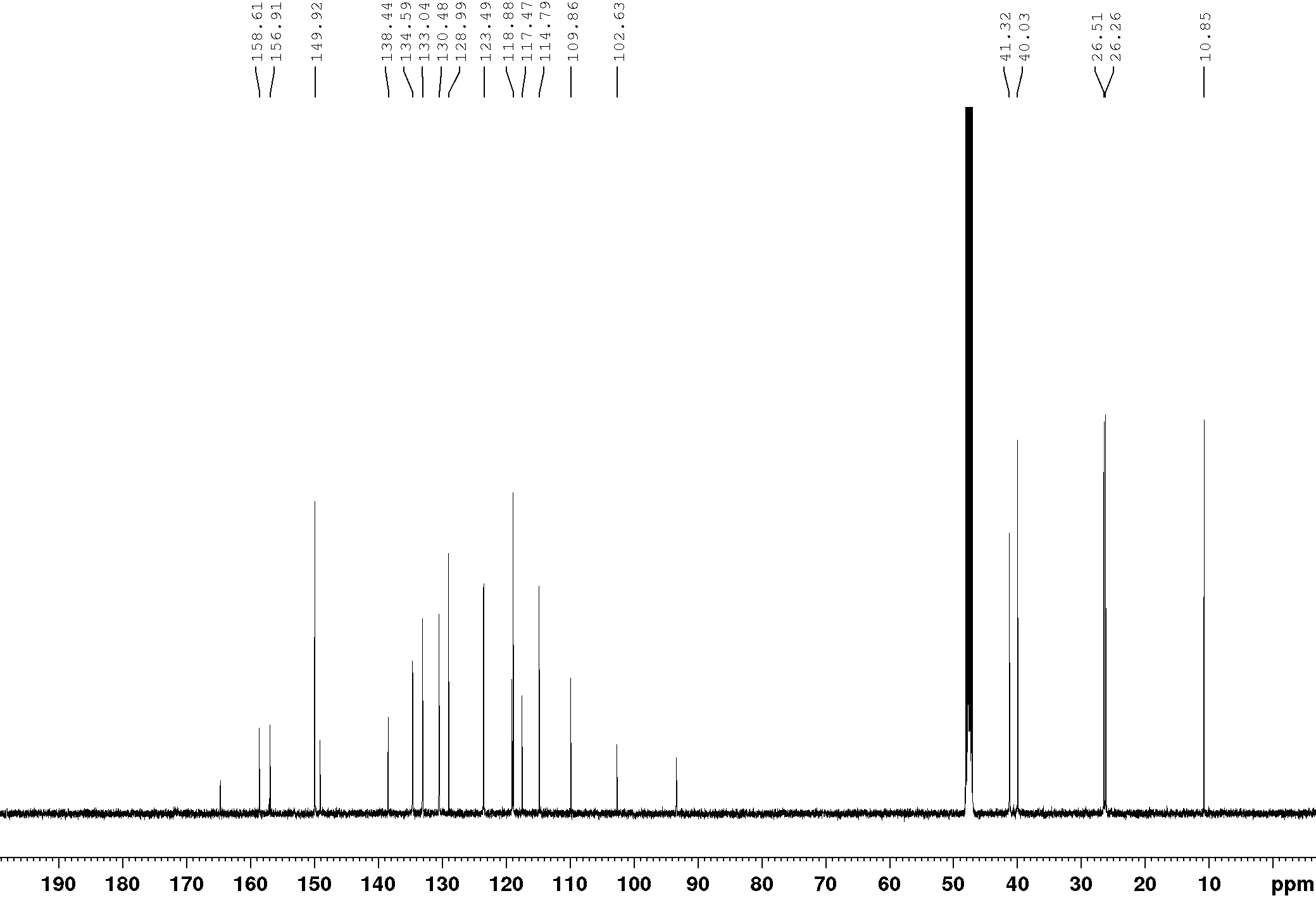


Analytical HPLC trace

HRMS ESI+ spectra

**1-(3-chlorophenyl)-2-cyano-3-((1*r*,4*r*)-4-((5-methyl-7*H*-pyrrolo[2,3-*d*]pyrimidin-4-yl)amino)cyclohexyl)guanidine (17).**

^1^H NMR (MeOD, 400 MHz)


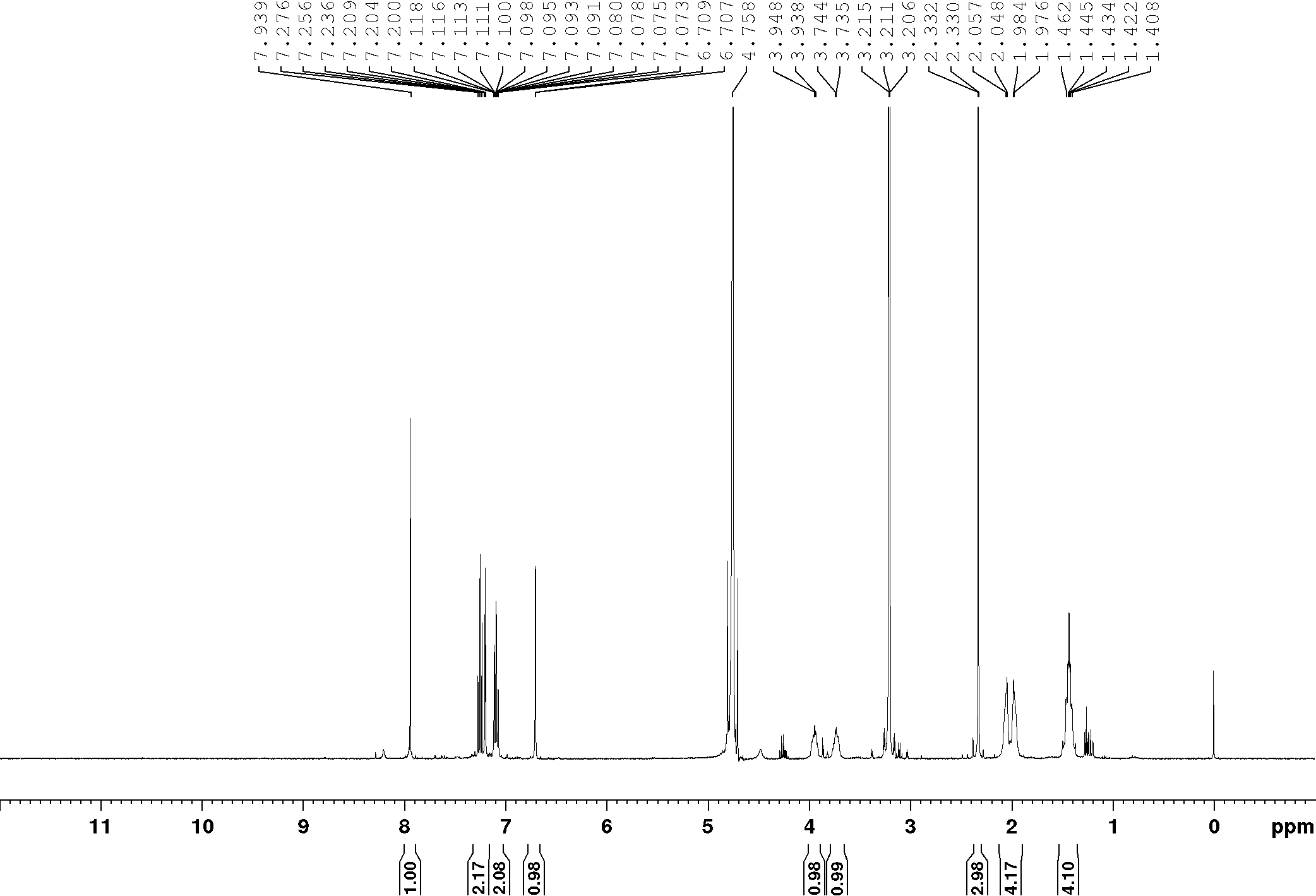


^13^C NMR (MeOD, 125 MHz)


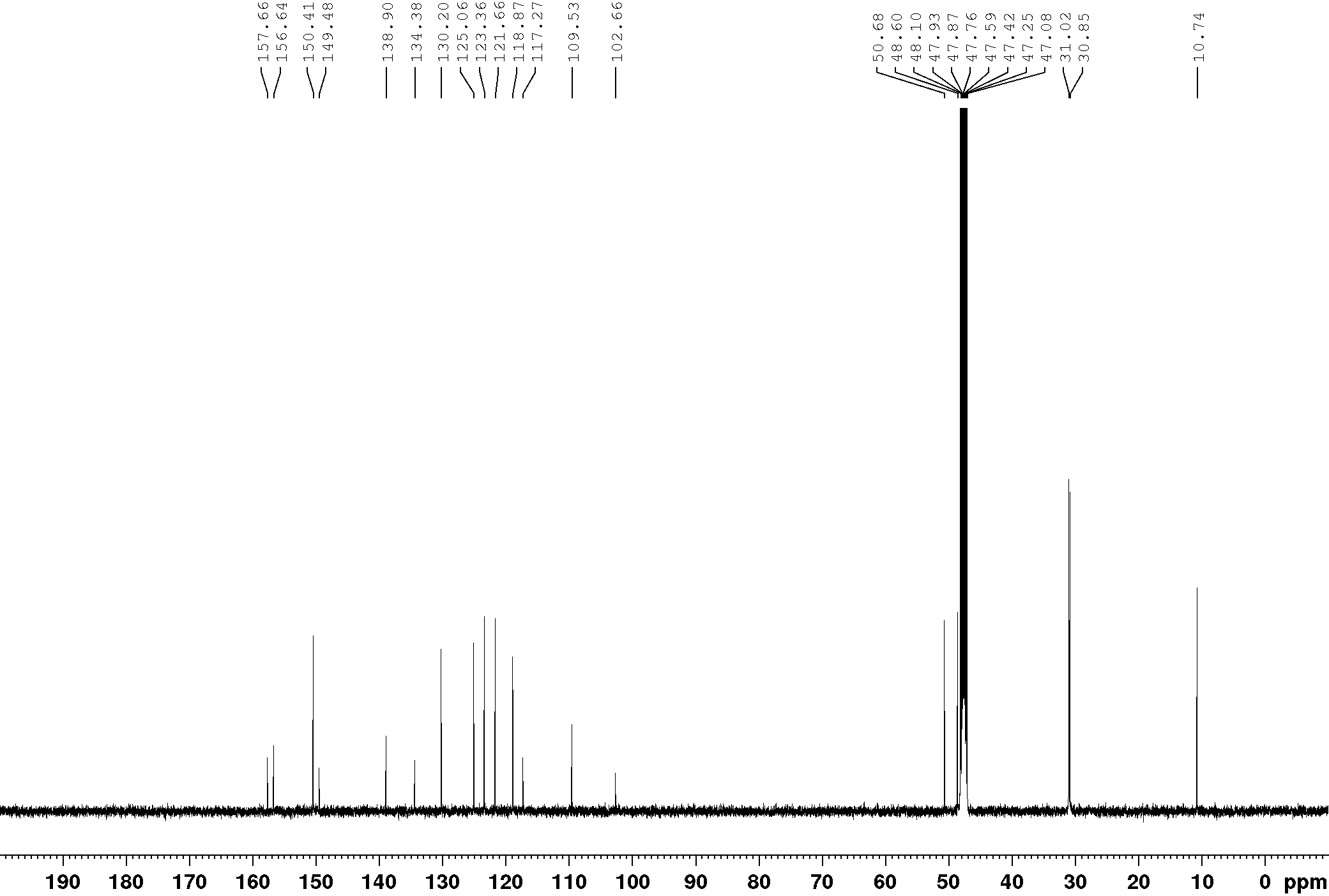


Analytical HPLC trace

HRMS ESI+ spectra

**1-(3-bromophenyl)-2-cyano-3-((1*r*,4*r*)-4-((5-methyl-7*H*-pyrrolo[2,3-*d*]pyrimidin-4-yl)amino)cyclohexyl)guanidine (18).**

^1^H NMR (MeOD, 400 MHz)


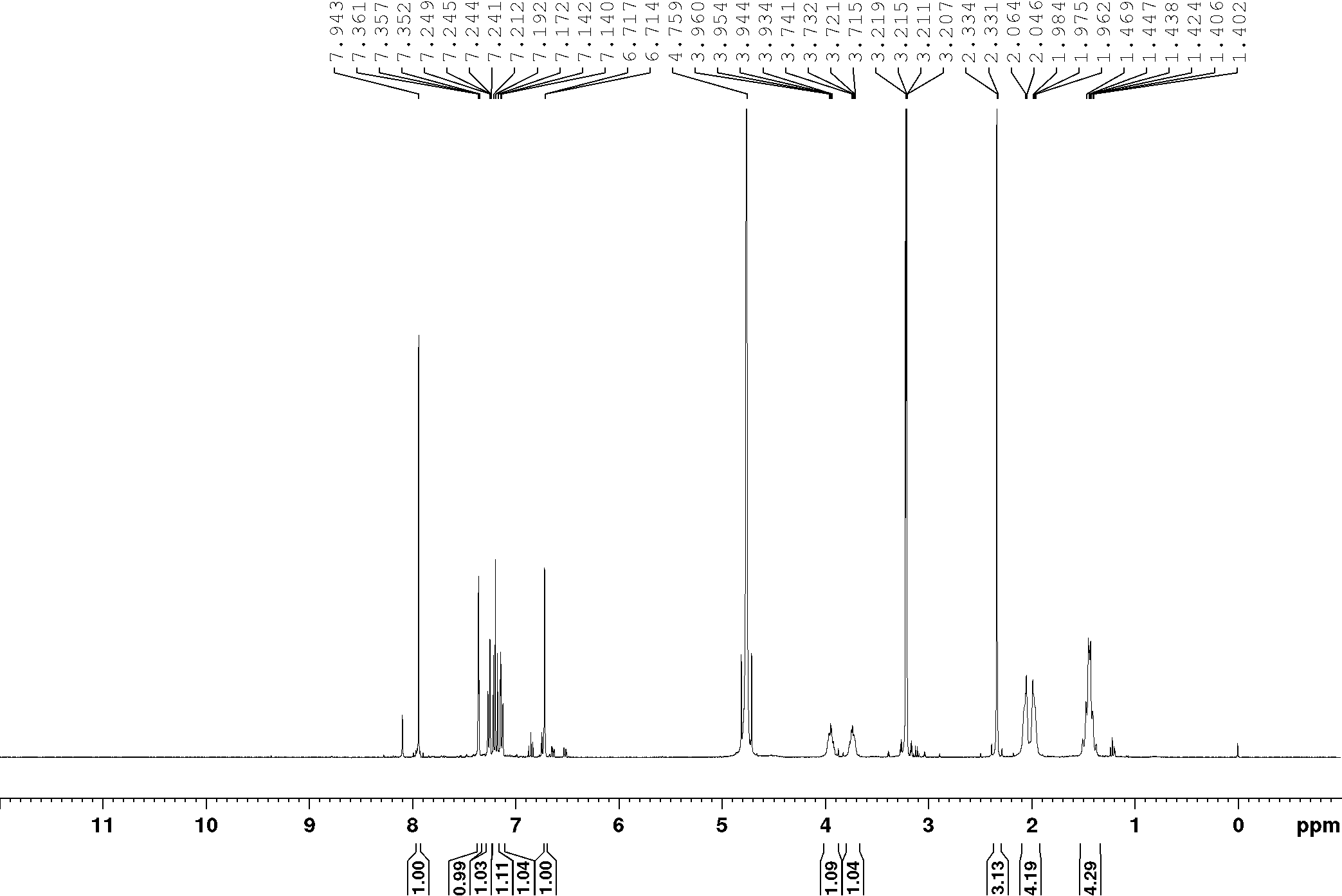


^13^C NMR (MeOD, 100 MHz)


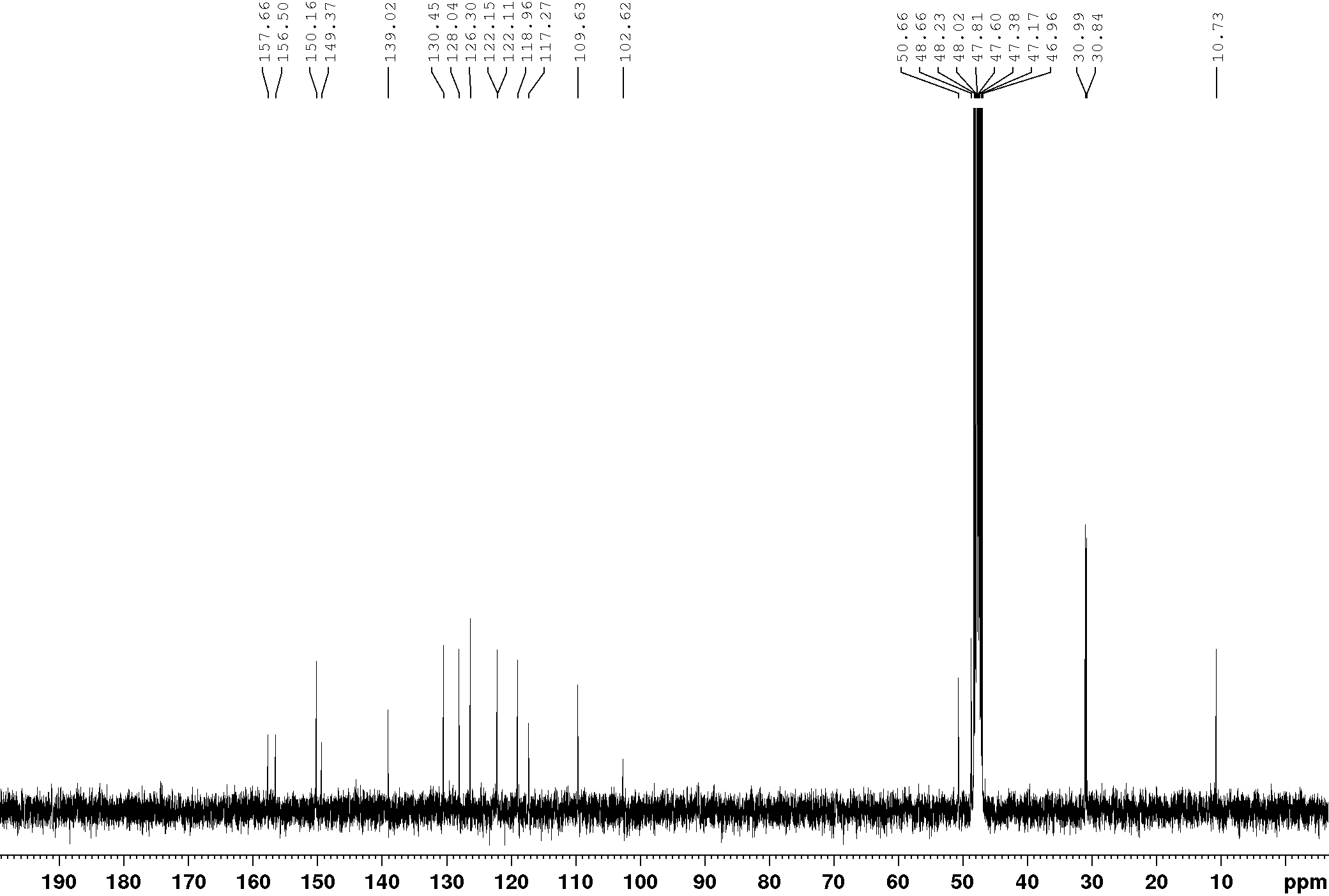


Analytical HPLC trace

HRMS ESI+ spectra

**2-cyano-1-(3-iodophenyl)-3-((1*r*,4*r*)-4-((5-methyl-7*H*-pyrrolo[2,3-*d*]pyrimidin-4-yl)amino)cyclohexyl)guanidine (19).**

^1^H NMR (MeOD, 400 MHz)


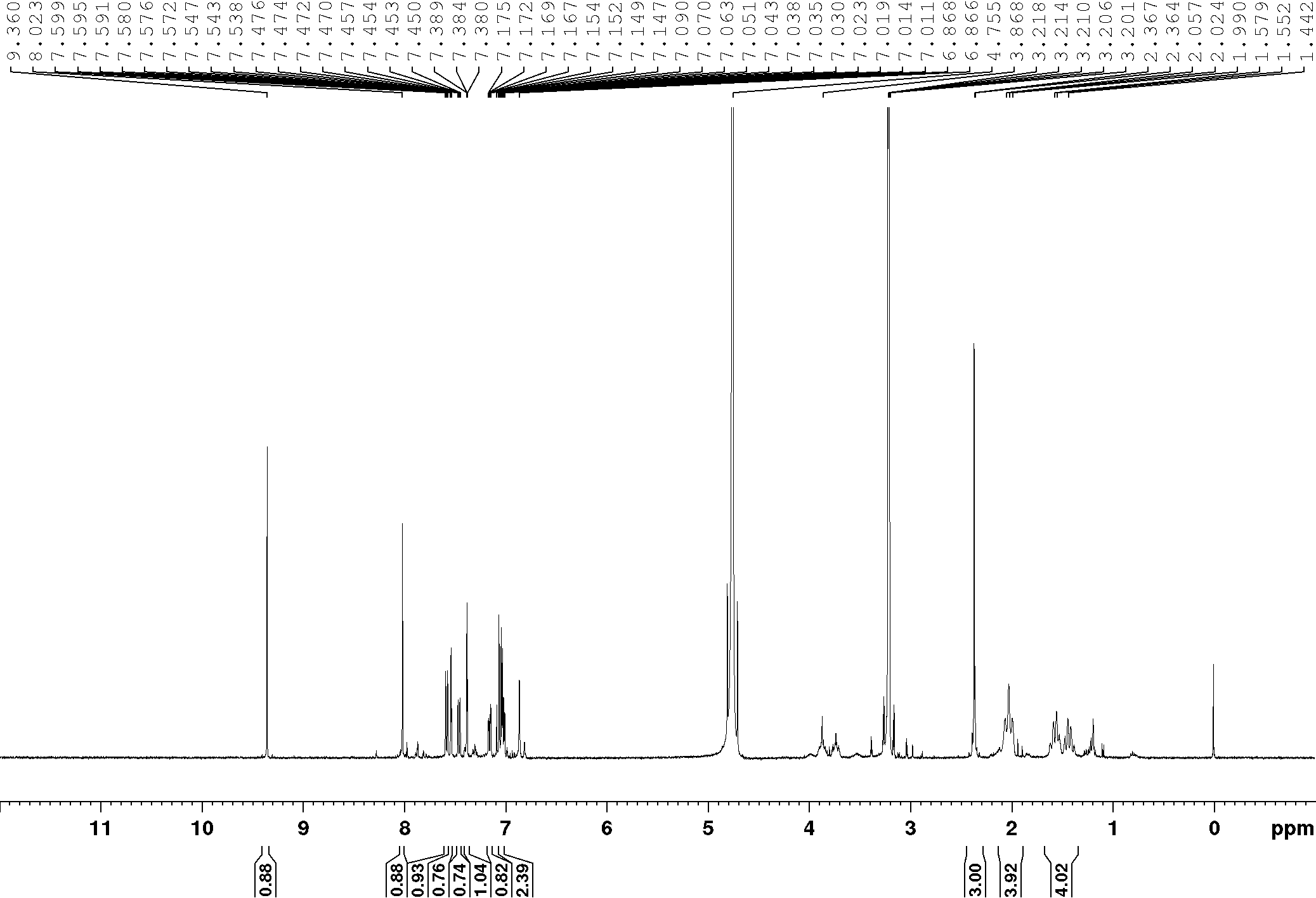


^13^C NMR (MeOD, 125 MHz)


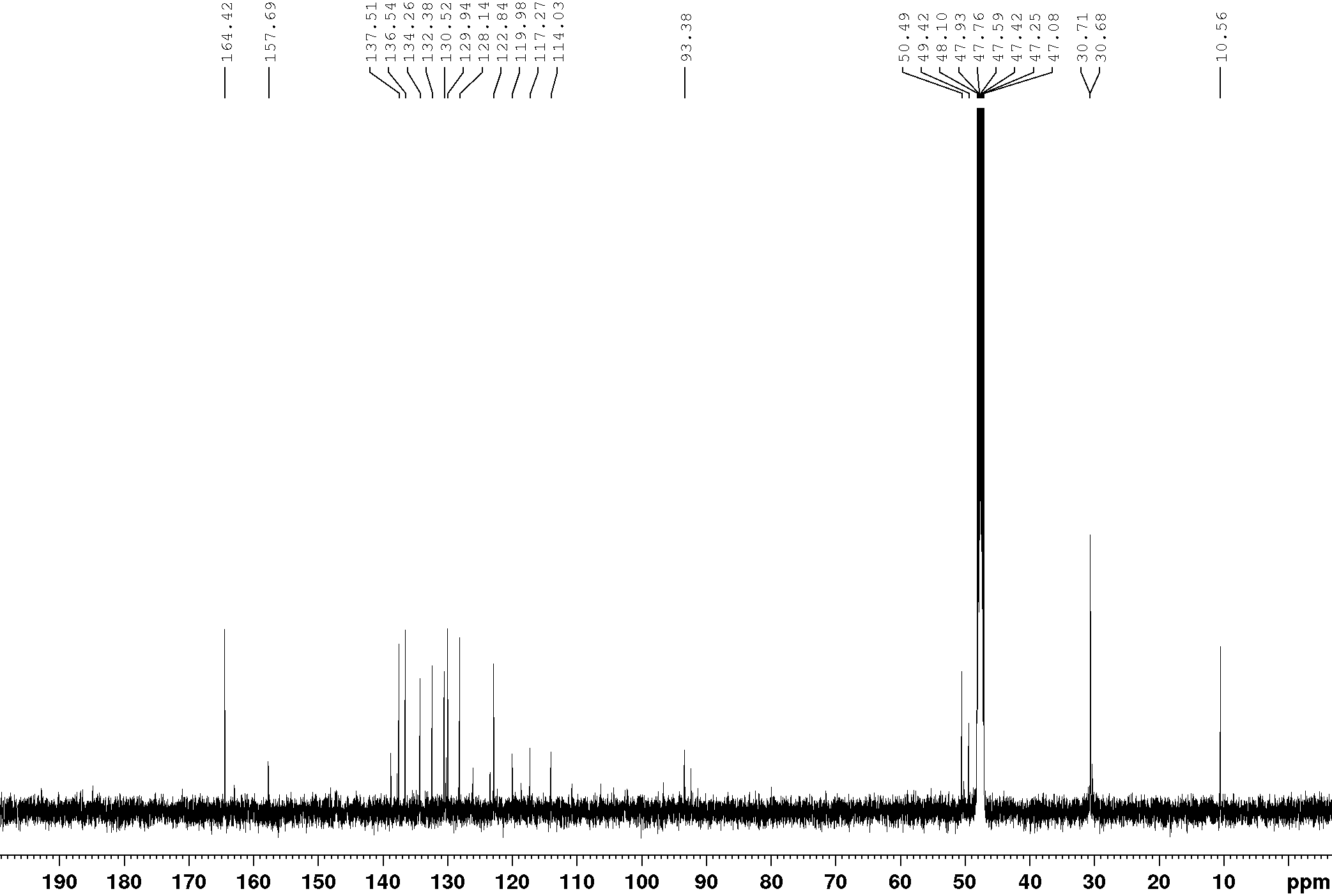


Analytical HPLC trace

HRMS ESI+ spectra

**(*S*)-*N*'-cyano-*N*-(3-iodophenyl)-2-methyl-4-(5-methyl-7*H*-pyrrolo[2,3-*d*]pyrimidin-4-yl)piperazine-1-carboximidamide (22).**

^1^H NMR (MeOD, 400 MHz)


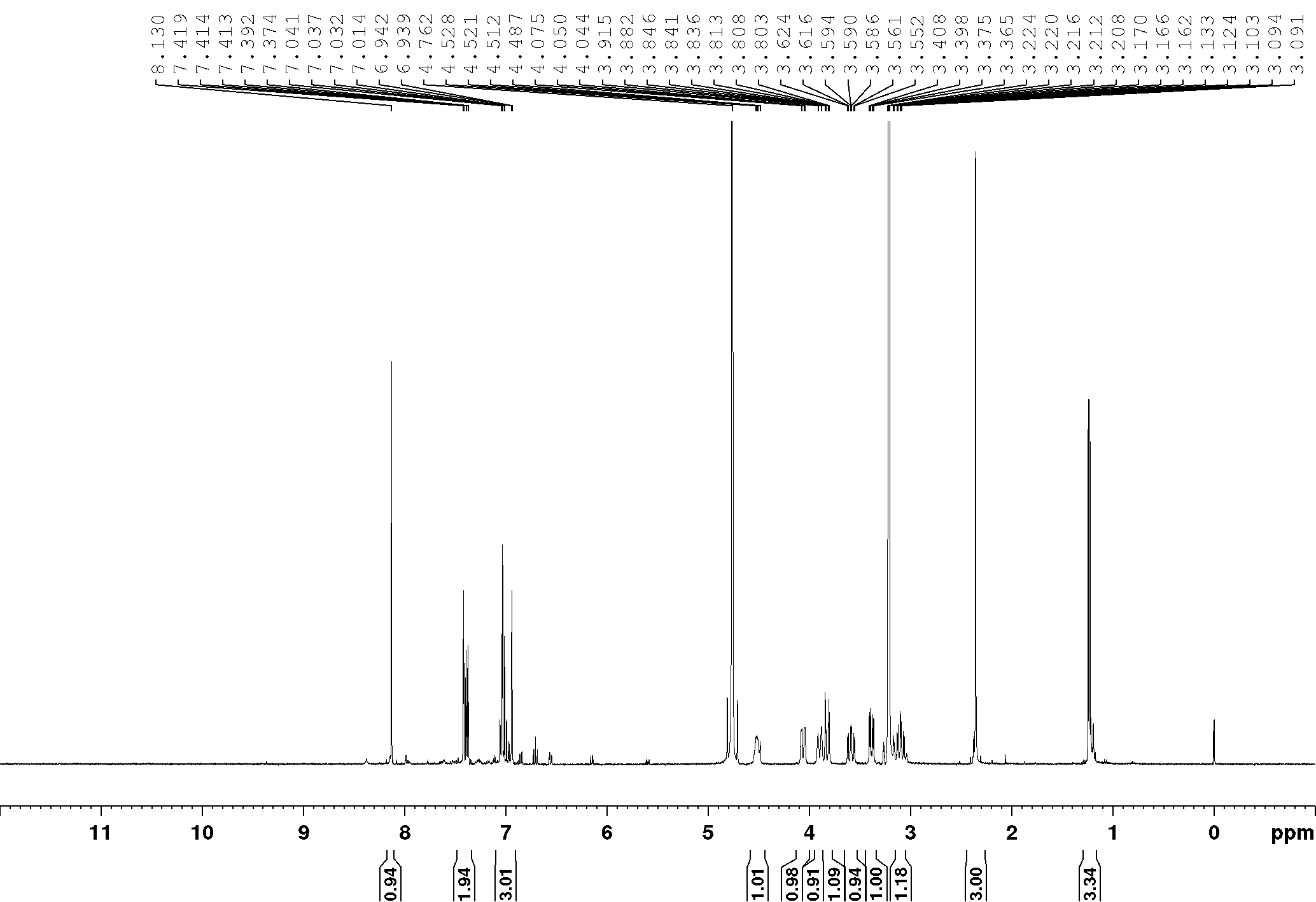


^13^C NMR (MeOD, 125 MHz)


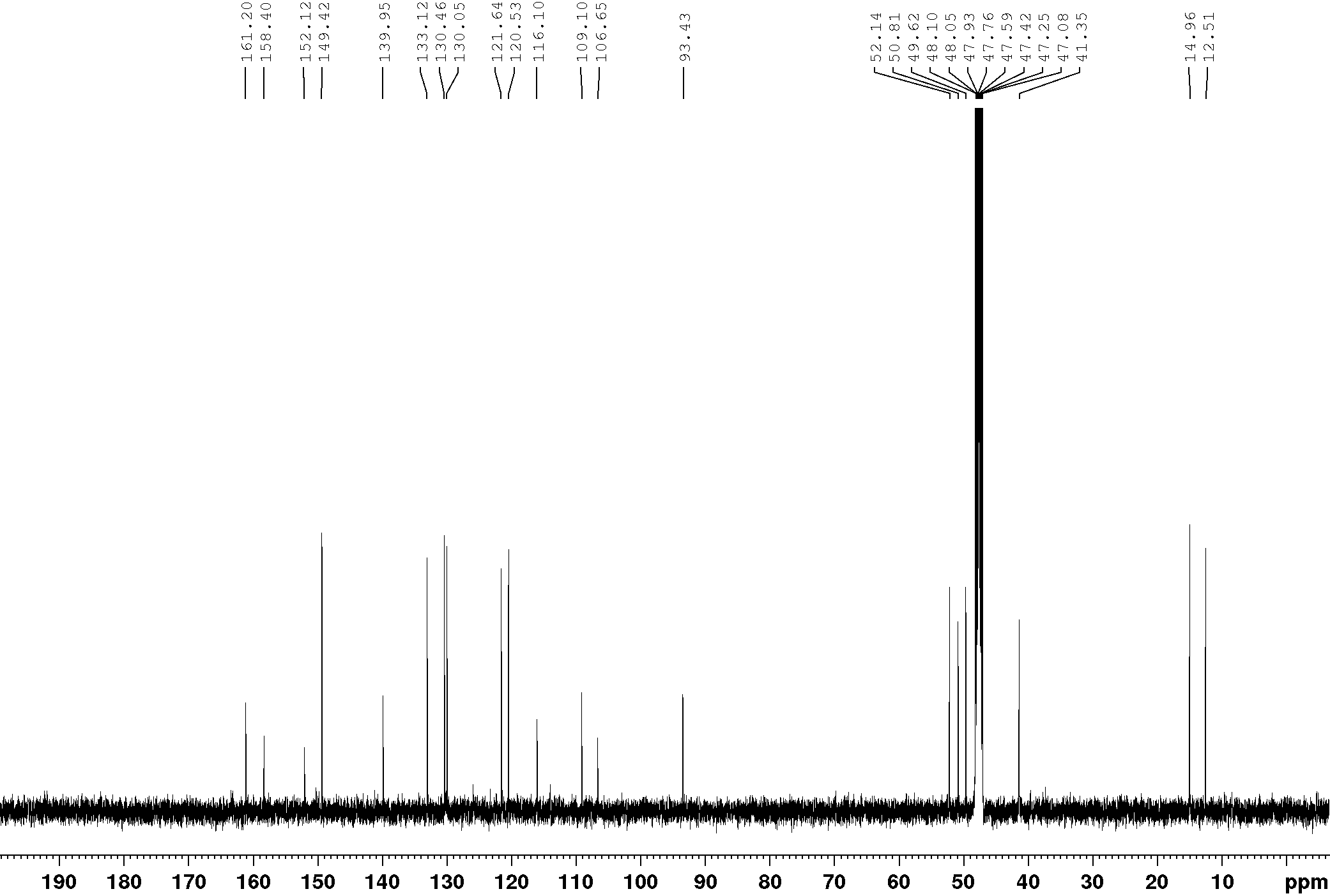


Analytical HPLC trace

HRMS ESI+ spectra

**1-(3-bromophenyl)-2-cyano-3-((1*R*,2*R*)-2-((5-methyl-7*H*-pyrrolo[2,3-*d*]pyrimidin-4-yl)amino)cyclohexyl)guanidine (23).**

^1^H NMR (MeOD, 400 MHz)


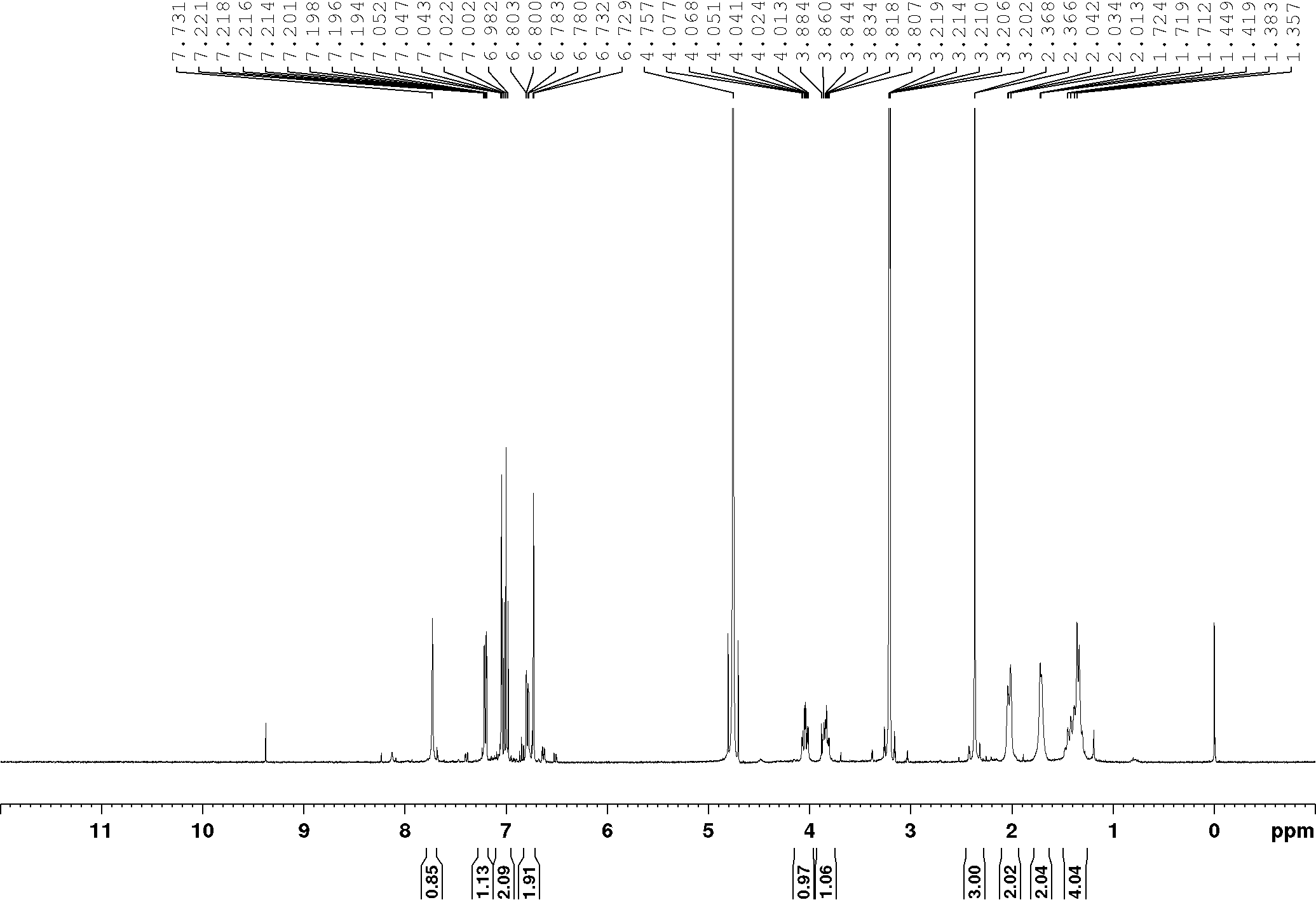


^13^C NMR (MeOD, 100 MHz)


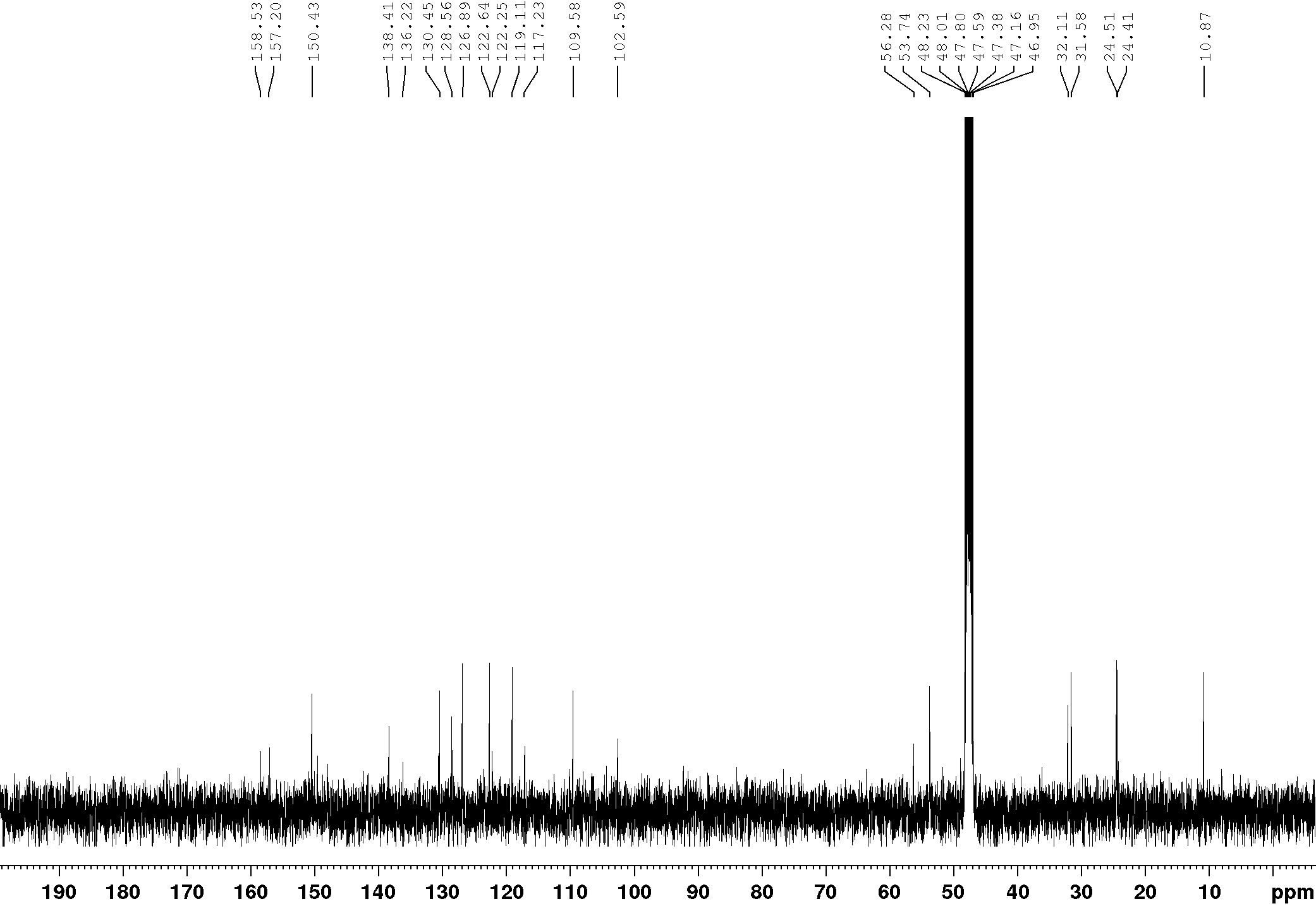


Analytical HPLC trace

HRMS ESI+ spectra

**2-cyano-1-(3-iodophenyl)-3-((1*R*,2*R*)-2-((5-methyl-7*H*-pyrrolo[2,3-*d*]pyrimidin-4-yl)amino)cyclohexyl)guanidine (24).**

^1^H NMR (MeOD, 400 MHz)


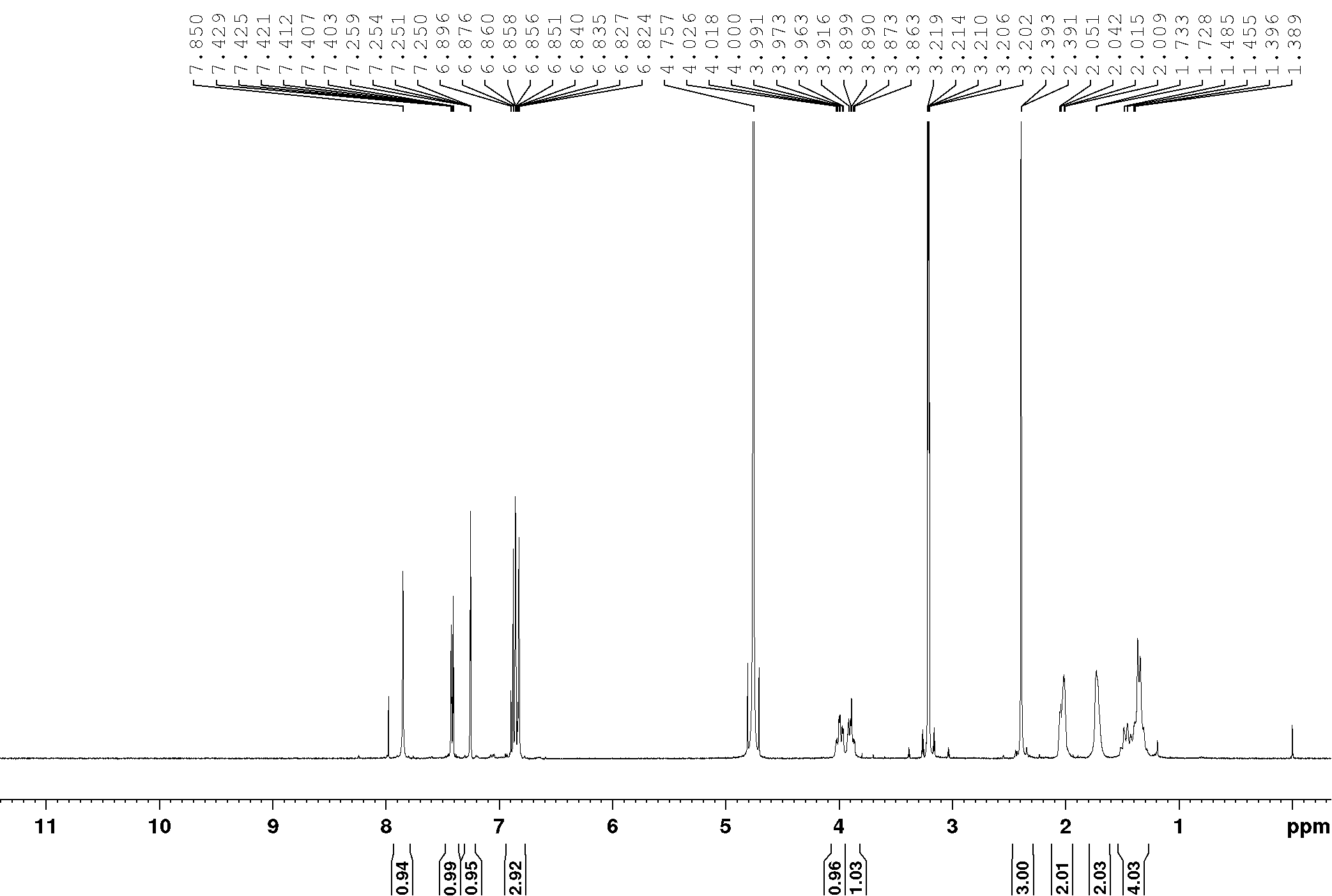


^13^C NMR (MeOD, 125 MHz)


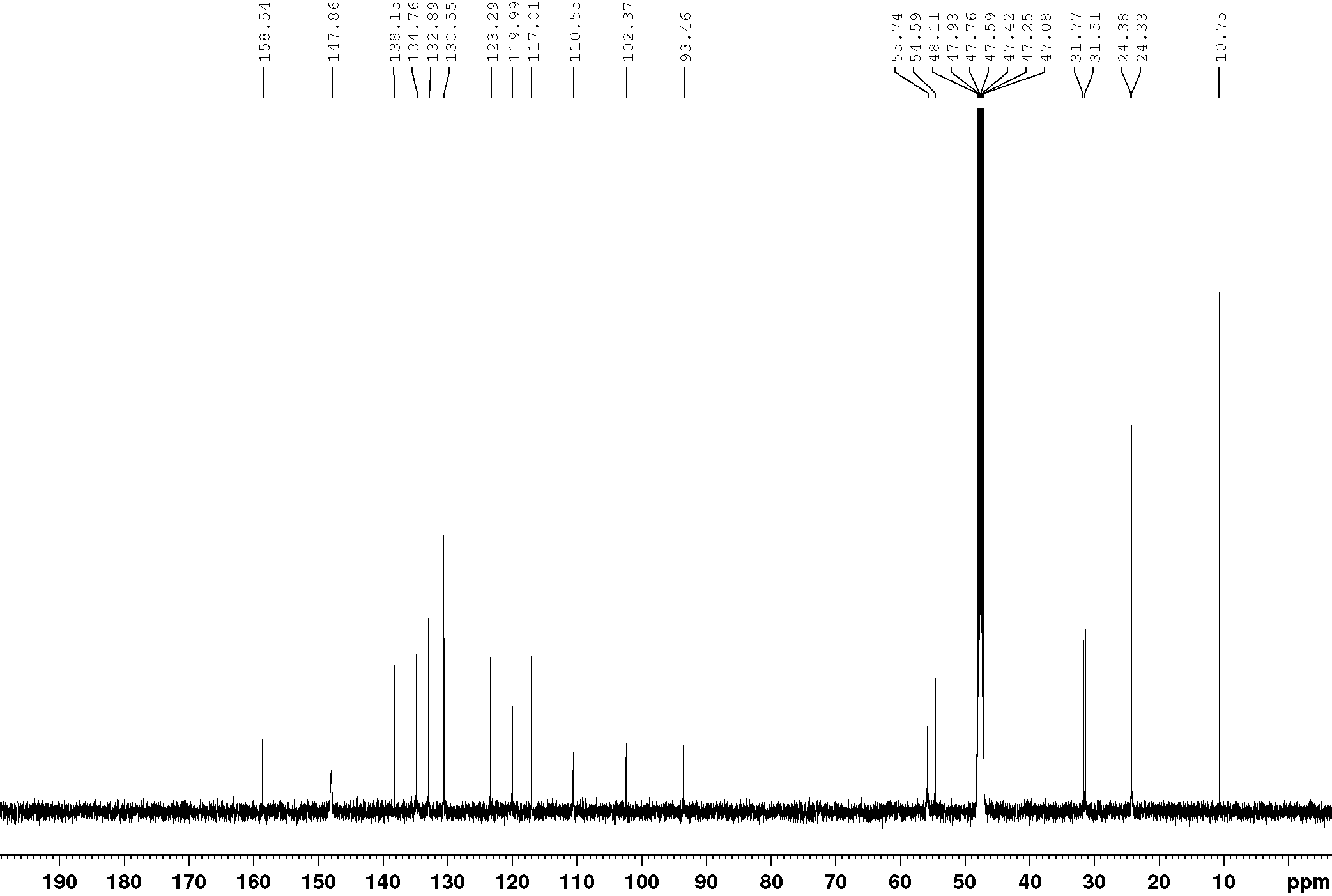


Analytical HPLC trace

HRMS ESI+ spectra

**1-(3-bromophenyl)-2-cyano-3-((1*S*,2*S*)-2-((5-methyl-7*H*-pyrrolo[2,3-*d*]pyrimidin-4-yl)amino)cyclohexyl)guanidine (25).**

^1^H NMR (MeOD, 400 MHz)


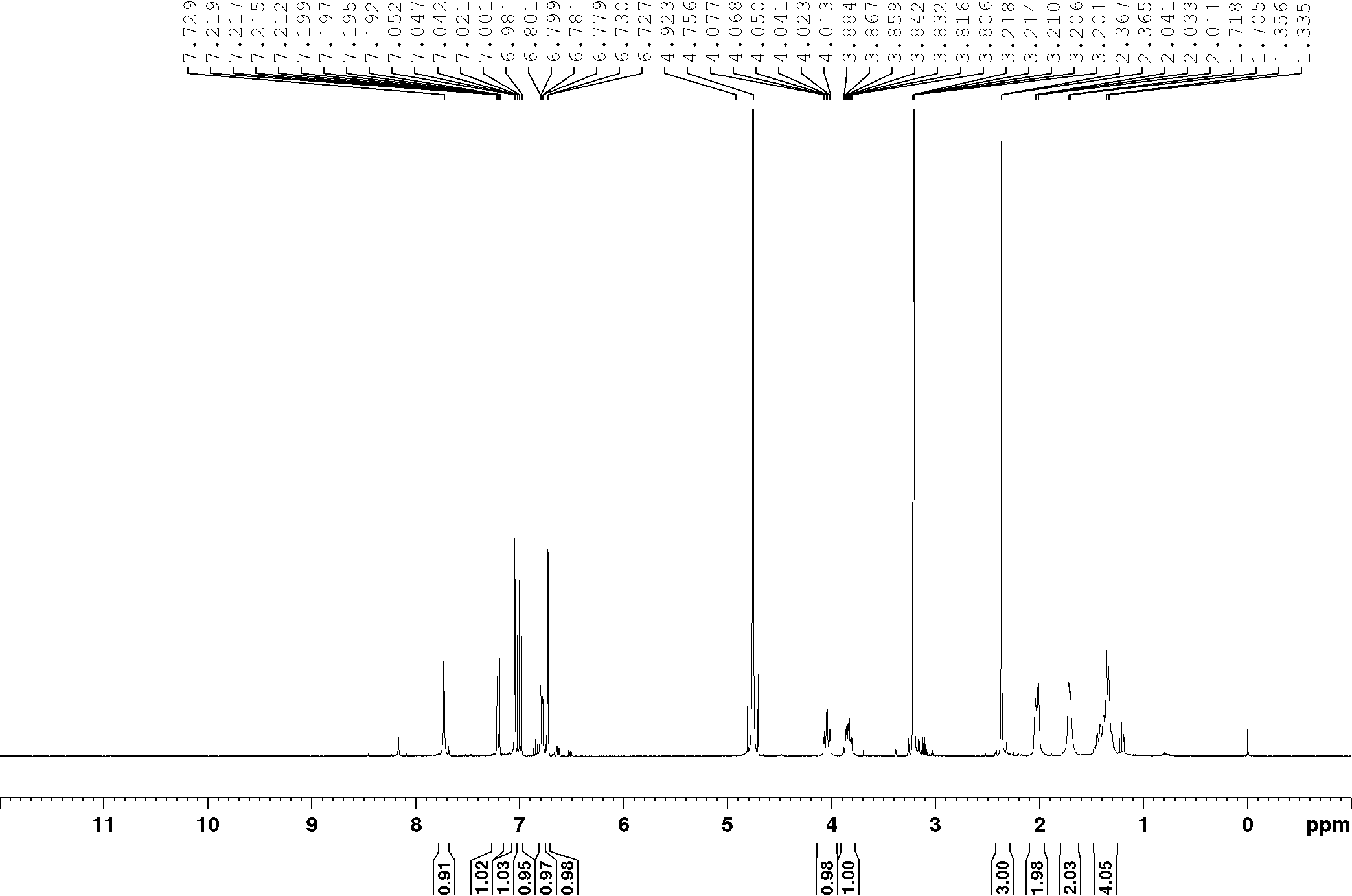


^13^C NMR (MeOD, 100 MHz)


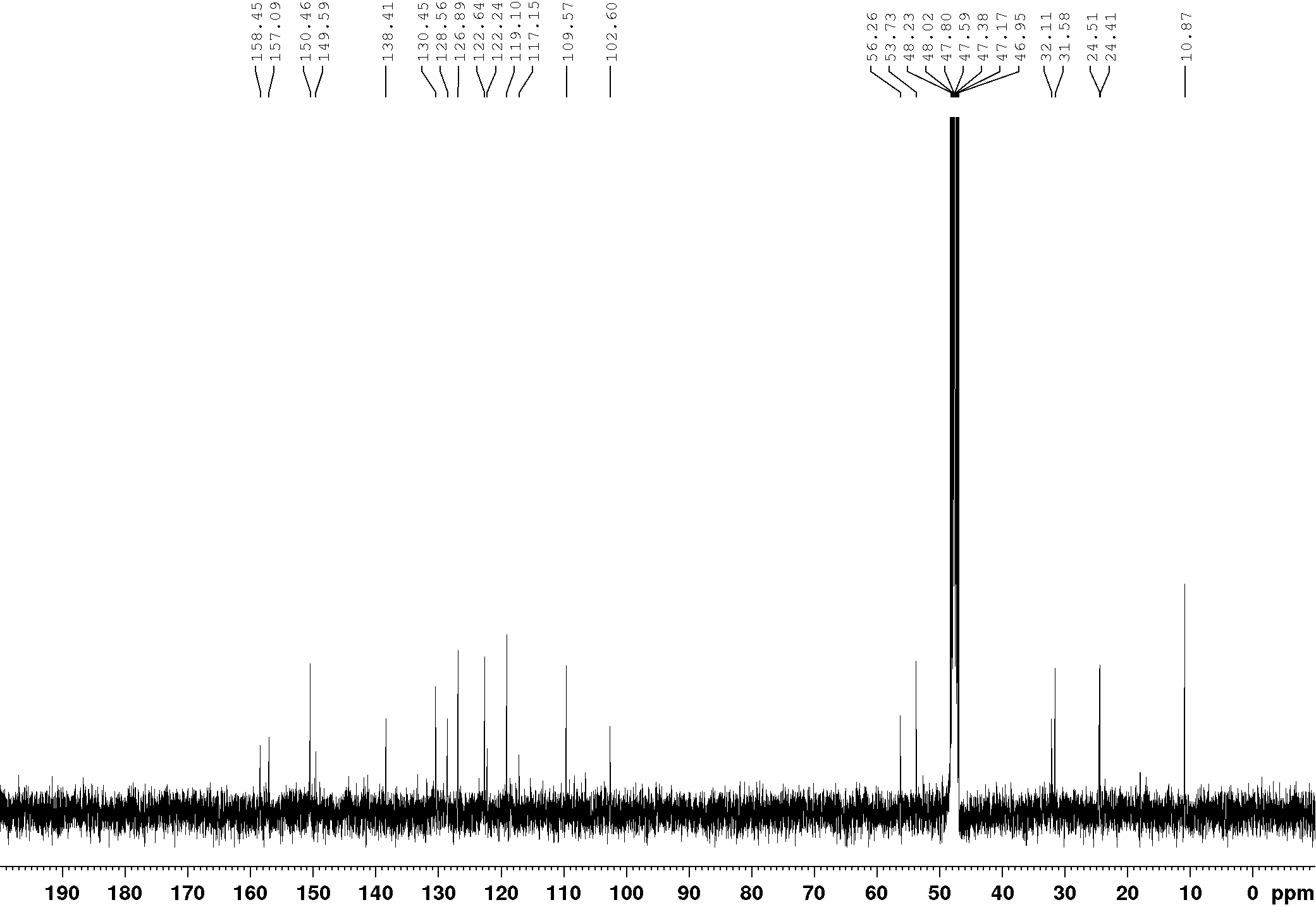


Analytical HPLC trace

HRMS ESI+ spectra

**2-cyano-1-(3-iodophenyl)-3-((1*S*,2*S*)-2-((5-methyl-7*H*-pyrrolo[2,3-*d*]pyrimidin-4-yl)amino)cyclohexyl)guanidine (26).**

^1^H NMR (MeOD, 400 MHz)


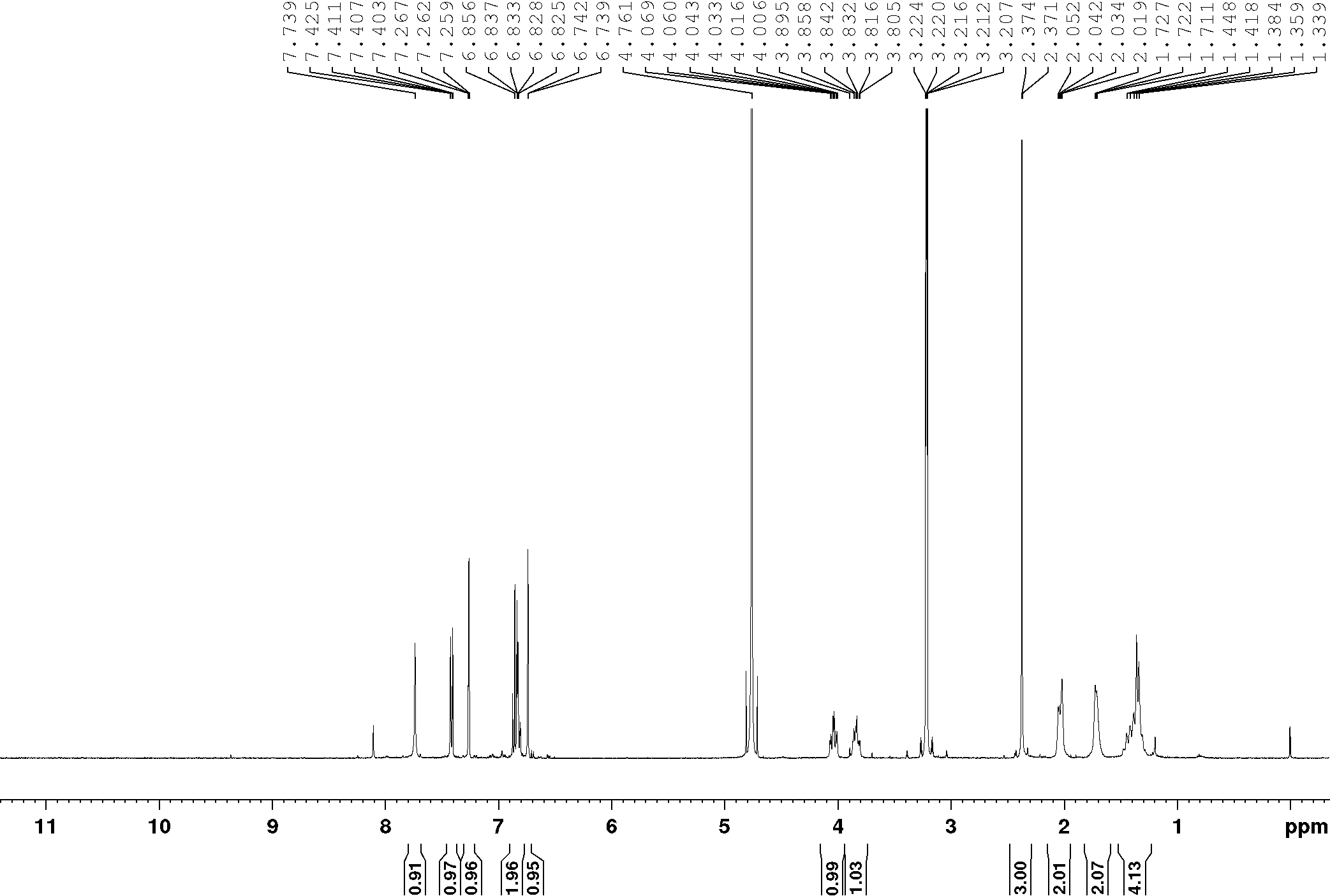


^13^C NMR (MeOD, 100 MHz)


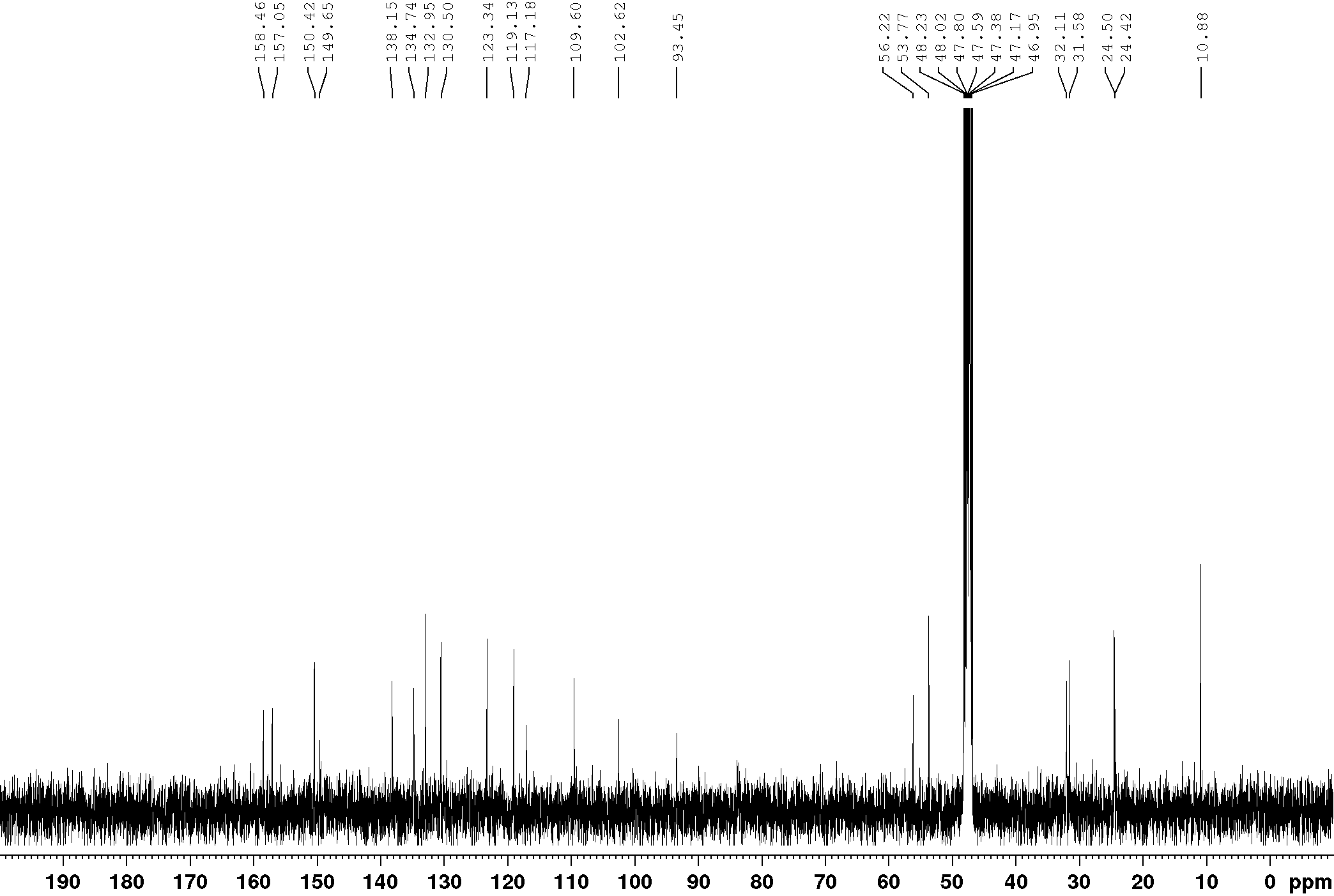


Analytical HPLC trace

HRMS ESI+ spectra

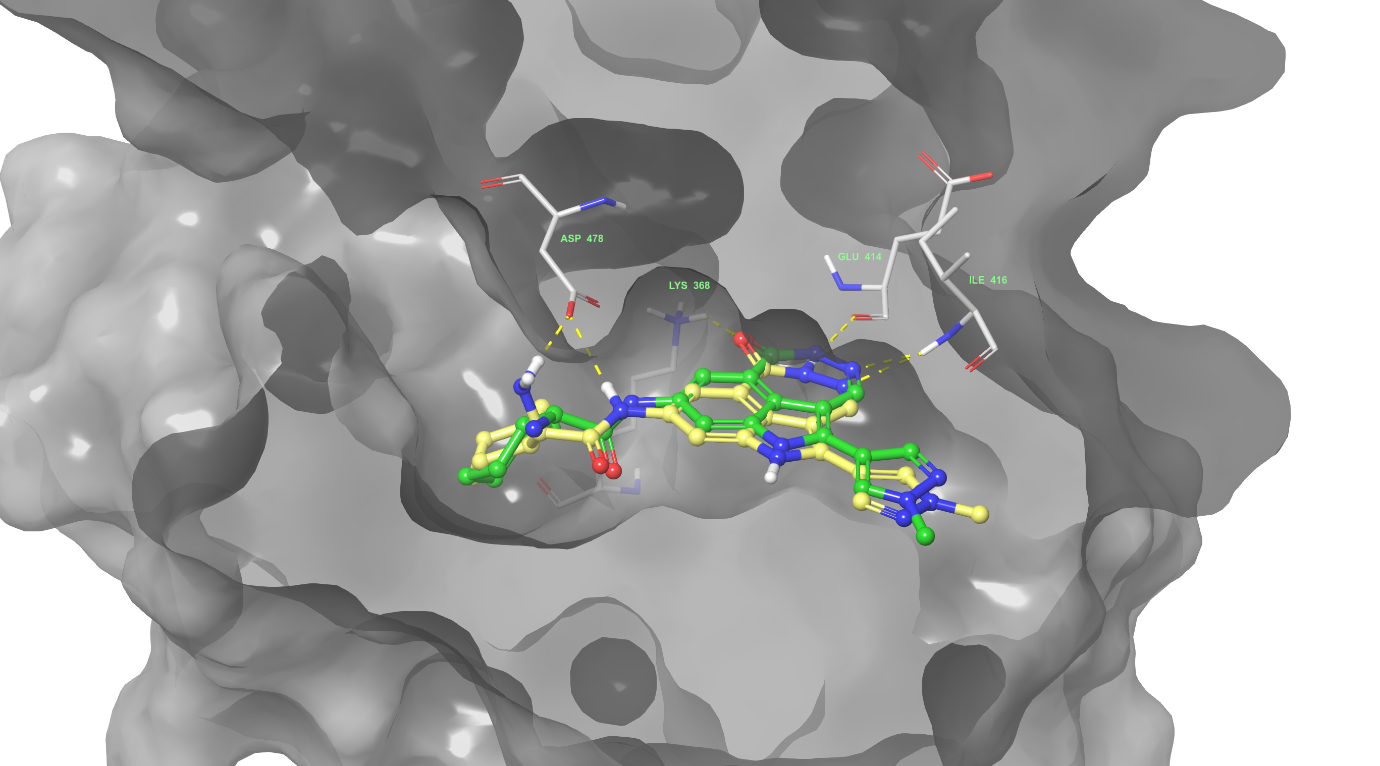


**Fig S1.** Comparison of LIMK1 crystal structure of 5NXC ligand (green) vs predicted binding pose (yellow). RMSD = 1.17 Å


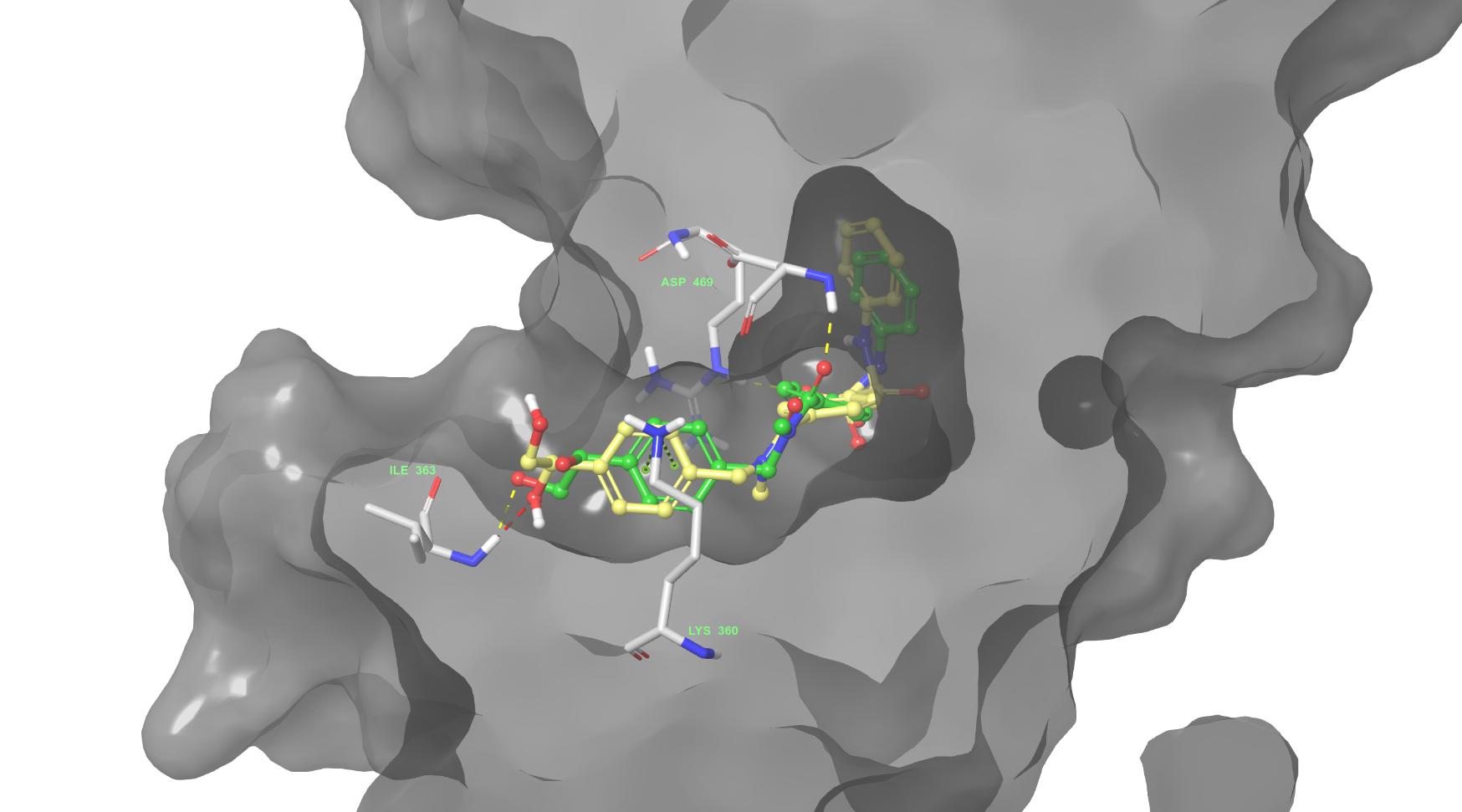


**Fig S2.** Comparison of LIMK1 crystal structure of 4TPT ligand (green) vs predicted binding pose (yellow). RMSD = 1.17 Å

**Table S1.** GLIDE score of compounds **1, 24** and **26** docking with LIMK1 and LIMK2

| Compounds | LIMK1 | LIMK2 |
| --- | --- | --- |
|  | –8.52 | –5.02 |
|  | –4.92 | –3.78 |
|  | –5.43 | –2.44 |
